# Deracemization of Carbohelicenes by a Chiral Perylene Bisimide Cyclophane Template Catalyst

**DOI:** 10.1002/anie.202104591

**Published:** 2021-06-03

**Authors:** Manuel Weh, Jessica Rühe, Benedikt Herbert, Ana‐Maria Krause, Frank Würthner

**Affiliations:** ^1^ Institut für Organische Chemie Universität Würzburg Am Hubland 97074 Würzburg Germany; ^2^ Center for Nanosystems Chemistry (CNC) Universität Würzburg Theodor-Boveri-Weg 97074 Würzburg Germany

**Keywords:** chirality transfer, cyclophanes, deracemization, dyes/pigments, template catalysis

## Abstract

Deracemization describes the conversion of a racemic mixture of a chiral molecule into an enantioenriched mixture or an enantiopure compound without structural modifications. Herein, we report an inherently chiral perylene bisimide (PBI) cyclophane whose chiral pocket is capable of transforming a racemic mixture of [5]‐helicene into an enantioenriched mixture with an enantiomeric excess of 66 %. UV/Vis and fluorescence titration studies reveal this cyclophane host composed of two helically twisted PBI dyes has high binding affinities for the respective homochiral carbohelicene guests, with outstanding binding constants of up to 3.9×10^10^ m ^−1^ for [4]‐helicene. 2D NMR studies and single‐crystal X‐ray analysis demonstrate that the observed strong and enantioselective binding of homochiral carbohelicenes and the successful template‐catalyzed deracemization of [5]‐helicene can be explained by the enzyme‐like perfect shape complementarity of the macrocyclic supramolecular host.

## Graphical abstract

An inherently chiral perylene bisimide cyclophane has been synthesized that shows high binding affinities for carbohelicene guests in organic solvents. The perfect shape complementarity of the host–guest system was utilized for the deracemization of conformationally labile [4]‐ and [5]‐helicenes.

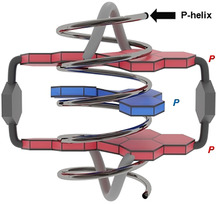

The field of supramolecular chemistry started with studies on macrocyclic receptors for the molecular recognition of cations and neutral molecules.[^1^, ^2^] For the latter, cyclophanes in particular enjoyed, and continue to enjoy, great popularity because they can provide suitable binding pockets to surround guest molecules and afford both binding strength and selectivity by shape complementarity.^[3]^ Accordingly, cyclophanes and related macrocycles have shown their usefulness for the complexation of various guest molecules,^[4]^ the construction of molecular machines,^[5]^ as molecular sensors,^[6]^ and for supramolecular catalysis.^[7]^ Whereas the earlier examples of synthetic cyclophane‐type supramolecular hosts typically were of high symmetry, recent studies have also explored the construction of cavities of lower symmetry for enantioselective recognition and sensing.[^8^, ^9^] Furthermore, Yashima and co‐workers recently reported double‐stranded spiroborate helicates that can be transformed into their optically active forms by the complexation of chiral guest molecules.^[10]^ However, to the best of our knowledge, despite the long history of research on the deracemization of organic molecules^[11]^ by dynamic kinetic resolutions via inclusion complexes^[12]^ and chromatography on chiral phases,^[13]^ deracemization by a templating chiral cyclophane host has not yet been demonstrated.

Toward this goal we considered our recently introduced perylene bisimide (PBI) based cyclophane hosts as particularly promising candidates. As a consequence of their large π‐surfaces, PBIs bridged with *para*‐xylylene spacer units proved to be excellent hosts with high binding affinities for polycyclic aromatic hydrocarbons[^14^, ^15^] and even some alkaloids.^[16]^ Furthermore, similar to previously described examples,[^8^, ^9^] chirality transfer from chiral guest molecules to achiral PBI cyclophanes could be observed by CD spectroscopy.^[17]^ Inspired by this work, we have now designed the first example of an inherently chiral PBI cyclophane host. As we will show, this host exhibits a very high binding affinity for [4]‐ and [5]‐helicene that can be utilized for the template‐catalyzed deracemization of the latter into an enantioenriched mixture with an enantiomeric excess (*ee*) of 66 %.

The key step in the synthesis of the inherently chiral PBI cyclophanes **1‐*MM*** and **1‐*PP*** (Schemes 1 and S1 in the Supporting Information) is an efficient intramolecular ring‐closing metathesis with the second‐generation Grubbs catalyst in 82 % yield, followed by the almost quantitative hydrogenation of the resulting olefinic double bond. In this way, stable atropisomers were obtained as a racemic mixture of *P*‐ and *M*‐enantiomers.[^18^, ^19^] The subsequent reaction sequence includes a saponification and imidization with (*R*)‐phenylethylamine to afford a highly soluble mixture of diastereomers that could be separated by chiral HPLC (Figures S25 and S26). Subsequent saponification and cyclophane synthesis with *para*‐xylylenediamine was accomplished following our previously reported route.^[14]^ For details on the experimental procedures and characterization of all new compounds, see the Supporting Information.

**Scheme 1:**
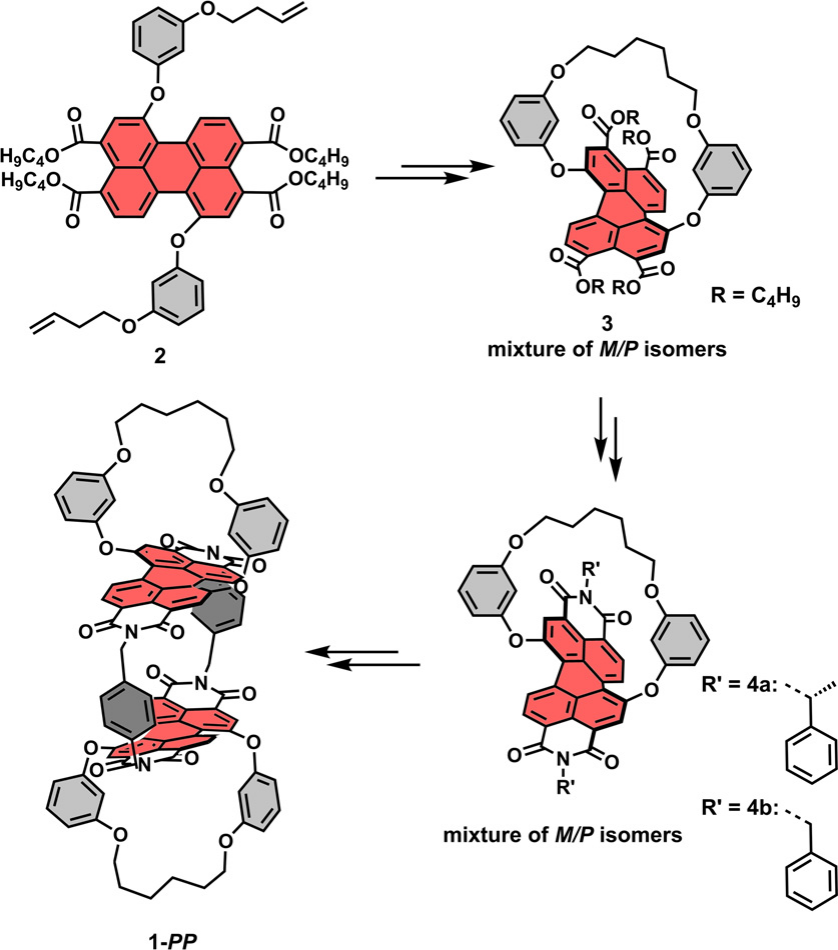
Synthetic route to PBI cyclophane **1‐*PP*** (the route to **1‐*MM*** is analogous) via diastereomer **4 a**. The structure of reference PBI dye **4 b** is also shown.

The properties of chiral cyclophanes **1‐*MM*** and **1‐*PP*** were studied by UV/Vis absorption, fluorescence, and CD spectroscopy in comparison to the reference dyes **4 a** and **4 b**. UV/Vis absorption spectroscopy (Figure 1 a, blue solid line and see also Figure S27b) reveals a H‐type coupling between the transition dipole moments of the S_0_→S_1_ transition of the two PBI units, which is manifested in a decrease in the *A*_0‐0_/*A*_0‐1_ ratio from 1.6 for the monomeric PBIs **4 a,b** (Figure S27a) to 1.1 for the cyclophanes.[^20^, ^21^] Furthermore, by comparison of the experimental CD spectra with structurally related enantiopure PBI chromophores,^[22]^ we were able to assign the absolute configuration of the isomerically pure atropisomers of PBI **4 a‐*M*** and **4 a‐*P*** that were utilized for the synthesis of **1‐*MM*** and **1‐*PP***. These results were further confirmed by time‐dependent density functional theory (TD‐DFT) calculations (Figure S39).

**Figure 1:**
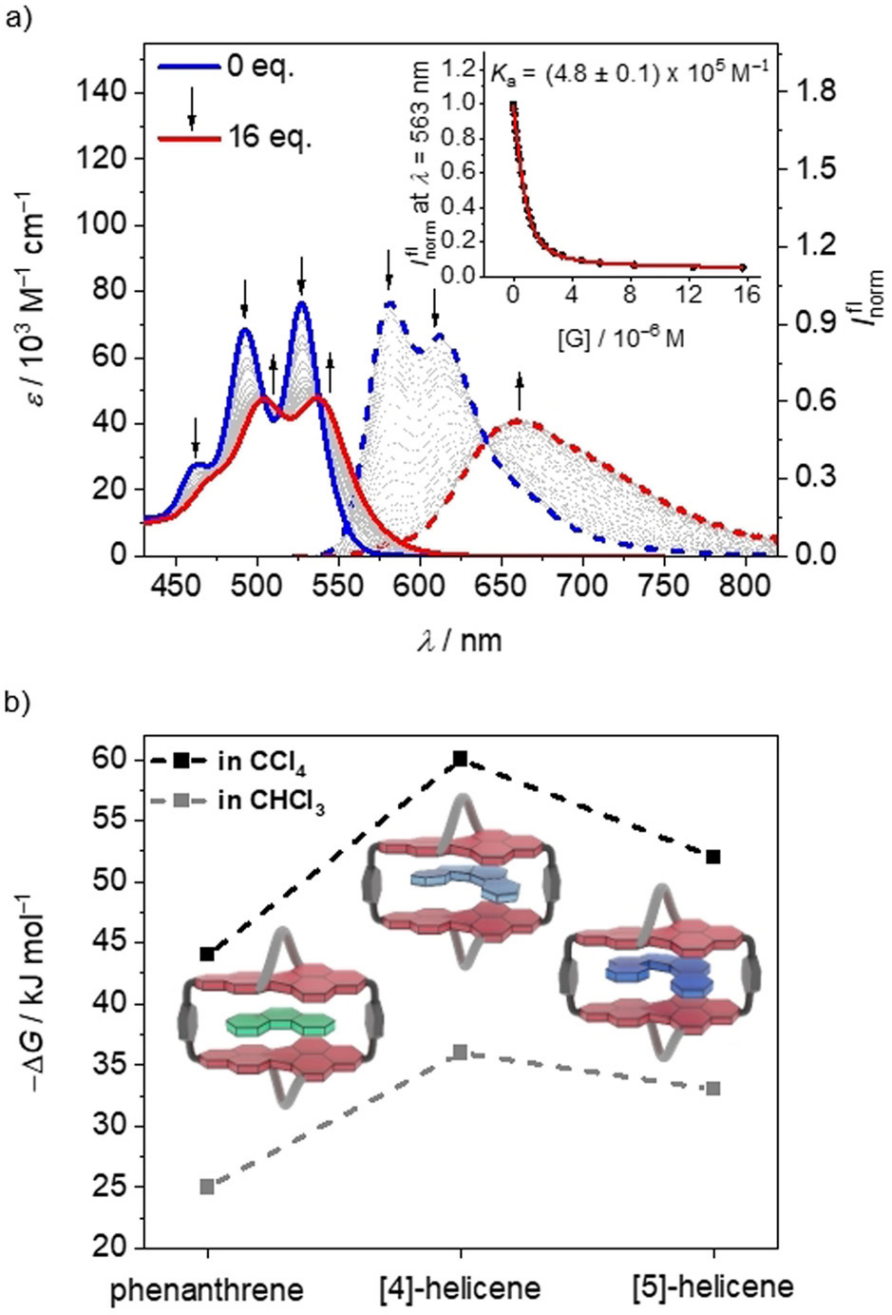
a) UV/Vis (solid lines) and fluorescence (dashed lines) titration curves of **1‐*PP*** in CHCl_3_ at 22 °C (*c*_host_=10 μm) upon the addition of *P*‐[5]‐helicene as a guest. Inset: Plot of the fluorescence intensity at *λ*=563 nm with a nonlinear curve fit (1:1 binding model, red curve). c) Plot of the Gibbs energy calculated by Δ*G*=−*RT*ln*K*_a_, with *T*=295 K and the respective binding constant.

Next, we were interested in the molecular recognition properties of these chiral macrocycles. Host–guest titration experiments were performed in chloroform using UV/Vis absorption and fluorescence spectroscopy. In our studies, we investigated the molecular recognition of a homologous series of carbohelicenes,[^23^, ^24^, ^25^] starting from the flat phenanthrene, which can be formally regarded as [3]‐helicene. The next congener, [4]‐helicene, is the first carbohelicene with a helical structure but cannot be isolated in enantiomerically pure form due to its low barrier for enantiomerization of only 17.2 kJ mol^−1^.^[26]^ Thus, [5]‐helicene is the first congener in this series for which the *M*‐ and *P*‐enantiomers can be resolved and whose racemization is sufficiently slow for binding studies. Figure 1 a presents the spectral changes of the UV/Vis absorption and fluorescence emission bands upon addition of *P*‐[5]‐helicene to a solution of **1‐*PP*** in chloroform at 22 °C. The spectral shifts in both experiments provide evidence for a significant charge‐transfer character, which affords a red‐shifted exciplex‐like emission band[^14^, ^27^] of lower intensity (Figure 1 a and Figure S33c) and an increased lifetime (Figure S28). More importantly, both titration studies with their well‐defined isosbestic and isoemissive points could be fitted with the 1:1 binding model^[28]^ to give *K*_a_=3.8×10^5^ m ^−1^ and *K*_a_=4.8×10^5^ m ^−1^, respectively. As expected, similar values, within experimental error, were obtained for the titration of **1‐*MM*** with *M*‐[5]‐helicene (Figure S34). From the average of these four titration experiments we derived a Gibbs energy of −32.1 kJ mol^−1^ for the 1:1 complex formed between the homochiral host and guest. For the heterochiral complexes, that is, **1‐*MM*** and *P*‐[5]‐helicene or **1‐*PP*** and *M*‐[5]‐helicene, binding was much weaker, but could not be evaluated because of an ongoing enantiomerization during the titration experiment (see below).^[29]^

Similar titration experiments for **1‐*MM*** (and **1‐*PP***) with phenanthrene and [4]‐helicene afforded averaged binding constants of *K*_a_=2.3×10^4^ m ^−1^ and *K*_a_=2.7×10^6^ m ^−1^ in chloroform at 22 °C, respectively (Figures S29–S32). Accordingly, the strongest binding is observed for [4]‐helicene, and we may assume that in this case host–guest complexes are formed in which the chiral information of the respective host has been imprinted on the guest to afford *P*‐[4]‐helicene⊂**1‐*PP*** and *M*‐[4]‐helicene⊂**1‐*MM***. Notably, no spectral changes were observed in the visible range for reference compound *rac*‐**4 b** upon the addition of [4]‐helicene, thus revealing no significant interaction between these molecules (Figure S35). Accordingly, the strong binding affinity observed for **1‐*MM*** and **1‐*PP*** arises from the encapsulation of the helicene guest between the two π‐faces.

As shown in Figure 1 b, significant increases in the binding affinities could be achieved for all carbohelicene guests in the less competitive solvent tetrachloromethane. Indeed, with a value of *K*_a_=3.9×10^10^ m ^−1^, the binding affinity for the complex with [4]‐helicene is in the nanomolar range and thus comparable to the binding affinity of modern drugs to their natural receptors. We note that such high binding constants can no longer be recorded by direct titration experiments and, therefore, had to be determined by competitive titration studies (Figures S36–S38).^[30]^

The structural features of the most strongly bound [4]‐helicene⊂**1‐*MM*** complex were elucidated by NMR spectroscopy and single‐crystal X‐ray crystallography. For the NMR experiment, we prepared a chloroform solution of **1‐*MM*** with an excess of [4]‐helicene and separated the excess guest by gel‐permeation chromatography (GPC) using chloroform as an eluent. Afterwards, ^1^H NMR spectroscopic analysis of the remaining mixture revealed a 1:1 host–guest stoichiometry, thereby corroborating not only the high binding affinity but also the kinetic stability of this complex against dissociation during the GPC separation. The protons of the free host and the complex were assigned by 2D NMR spectroscopy (Figures S41 and S42). A comparison of the ^1^H NMR spectrum of the host–guest complex with the free host in 1,1,2,2‐tetrachloroethane‐*d*_2_ (TCE) reveals an upfield shift of the perylene receptor protons (marked in red and orange), which is in accordance with a complexation of the carbohelicene inside the cavity (Figure 2 a). Furthermore, both the methylene protons (marked in turquoise) and the aromatic protons of the xylylene spacer (marked in green) experience a downfield shift caused by the aromatic ring current of the helicene guest.

**Figure 2:**
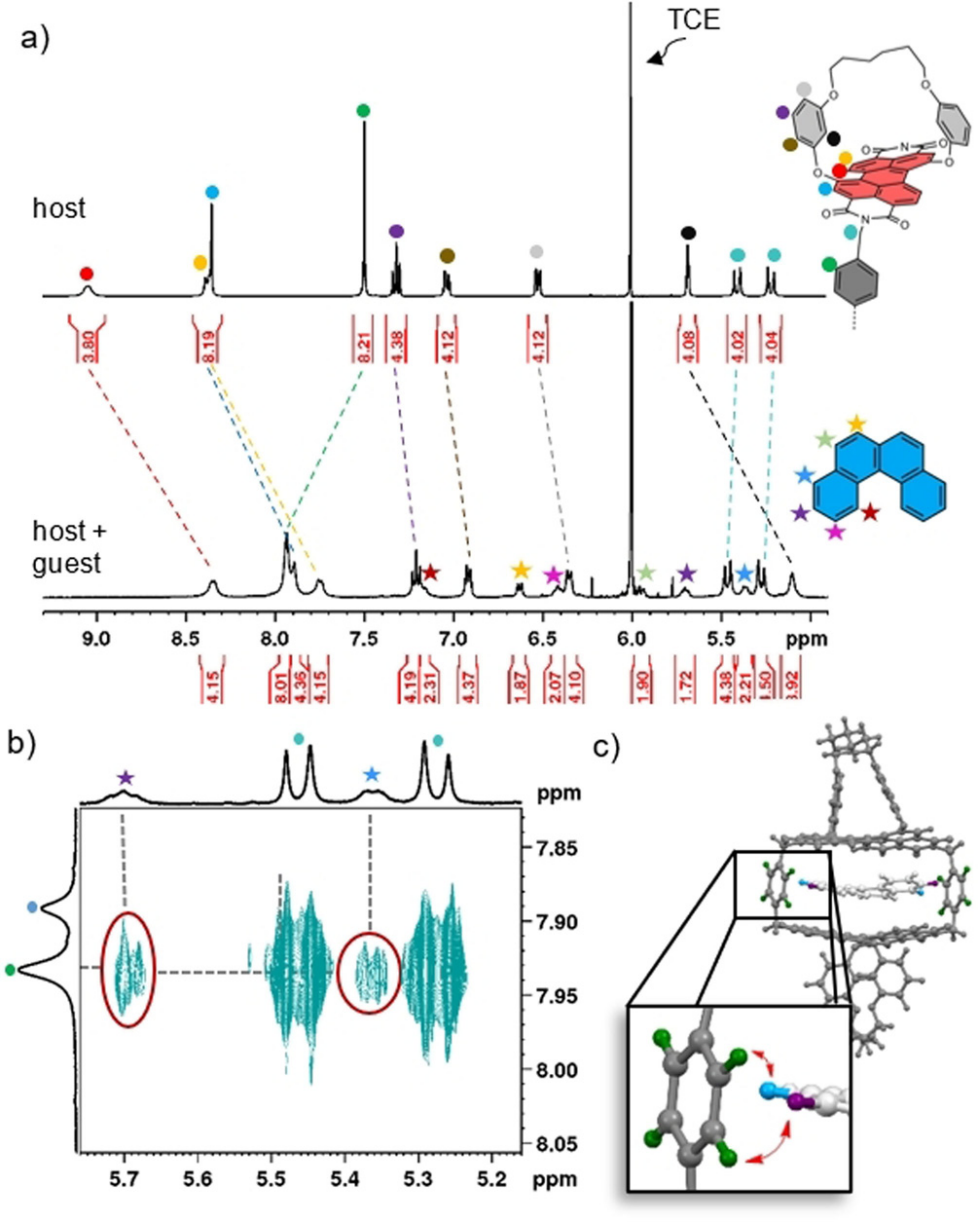
a) Partial 400 MHz ^1^H NMR spectrum of the free host **1‐*MM*** (*c*=3.5 mm) and of [4]‐helicene⊂**1‐*MM*** (1 equiv [4]‐helicene, bottom) in TCE‐*d*_2_ at 298 K, including the assignment of the protons. The filled circles mark the protons of the host and the stars indicate the protons of the guest. b) Excerpt from a 400 MHz ^1^H‐^1^H ROESY NMR spectrum of [4]‐helicene⊂**1‐*MM*** in TCE‐*d*_2_ at 298 K with relevant cross‐signals. c) DFT‐optimized structure of [4]‐helicene⊂**1‐*MM*** with marked protons that show cross‐signals in the ROESY NMR spectrum.

To gain further insight into the arrangement of the guest within the cavity, a ^1^H‐^1^H ROESY NMR experiment was performed (Figures 2 b and S42b). This enables through‐space correlations between the protons of [4]‐helicene and the aromatic spacer protons to be observed, which can only be explained by the spatial proximity of these units. Our DFT‐optimized structure of [4]‐helicene⊂**1‐*MM*** is in accordance with this observation (Figures 2 c and S40). Alternative binding modes, such as the complexation of the guest with the outer part of the PBI can clearly not explain these signals.

The ultimate proof for the encapsulation of a homochiral *M*‐[4]‐helicene within the **1‐*MM*** cyclophane host, that is, molecular imprinting of chirality,^[31]^ could be accomplished by co‐crystallization of these molecules from a chlorobenzene/*n*‐hexane solution and subsequent single‐crystal X‐ray analysis (Figure 3, Figure S43, and Table S1). The *M*‐[4]‐helicene⊂**1‐*MM*** complex crystallizes in the monoclinic crystal system (space group *P*2_1_) with two complexes per unit cell. The solid‐state structure reveals that the macrocycle forms a box‐like cavity, which offers an ideal distance of about 7.2 Å between the perylene units for the encapsulation of polycyclic aromatic hydrocarbons through aromatic π‐π interactions. As shown in Figure 3 (and in more detail in Figure S43b), the two PBI moieties of the macrocycle have a parallel arrangement without rotational or longitudinal displacements. Furthermore, the core twist of the perylene chromophores, with an average dihedral angle between the naphthalene subunits of 15.4°, appears to be perfect for the accommodation of the homochiral *M*‐[4]‐helicene guest molecule. Accordingly, the structure of *M*‐[4]‐helicene embedded within **1‐*MM*** in this single crystal is in very good agreement with the DFT‐calculated structure of *M*‐[4]‐helicene (Figure S40). A further contribution to the strong binding of this guest molecule might arise from additional CH⋅⋅⋅π interactions (Figure 3, inset)^[32]^ between the aromatic spacer units of the host and the protons of the guest at a distance of 2.6–2.9 Å (the range indicates the different distances observed for the two sides in the cavity) in the [4]‐helicene⊂**1‐*MM*** complex. Notably, despite the excellent embedding of the guest by four sides, the crystal structure reveals a good accessibility of guest molecules to the cavity, which will be of relevance for the studies reported next.

**Figure 3:**
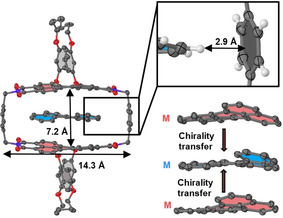
a) Molecular structure of the complex *M*‐[4]‐helicene⊂**1‐*MM*** obtained by single‐crystal X‐ray analysis. Hydrogen atoms are omitted for clarity. The perylene core is colored in red and the guest in blue. An enlarged excerpt of the host–guest complex is shown to illustrate the CH⋅⋅⋅π interactions between the guest and the xylylene spacer groups. For this purpose, the hydrogen atoms are displayed. In addition, an enlarged view of the perylene units of the host and the guest is shown to illustrate the homochirality of the host and guest.

As discussed above, our binding studies provided strong evidence for the enantioselective binding of helicene guest molecules that have a homochiral backbone to the host. Motivated by the large Gibbs energy observed for these complexes, we conceived an experiment for the deracemization of [5]‐helicene, whose racemization barrier of 100.8 kJ mol^−1^ corresponds to a process that takes place within about one day at room temperature (Figure S46b).[^26^, ^33^] Accordingly, a 1:1 mixture of chiral host and racemic guest was prepared in chloroform solution and the optical changes were followed over time by CD spectroscopy. The time‐dependent data indicate that the complexation takes place successively until an equilibrium between the two enantiomers of the guest, the complex, and the free host is reached (Figures 4 a and S45). An increase in the CD signal in the characteristic absorption range of [5]‐helicene (300–350 nm) is observed, which suggests the preferential molecular recognition of one carbohelicene enantiomer by the chiral host. However, a quantitative evaluation of the chirality transfer leading to deracemization of [5]‐helicene is hampered in this experiment by the significant overlap of the helicene absorption band with those of the PBI host (**1‐*MM*** or **1‐*PP***).

**Figure 4:**
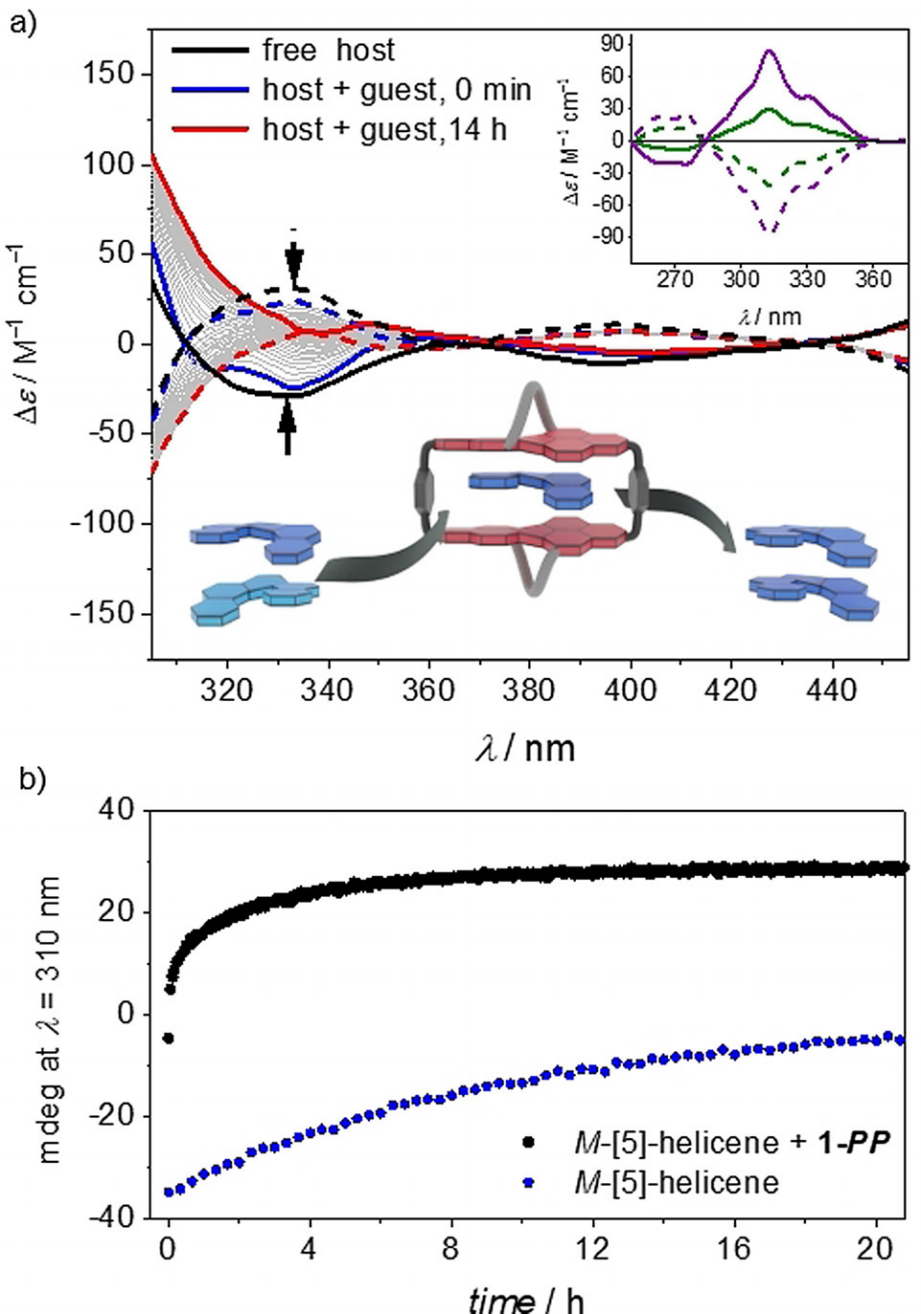
a) CD absorption spectra of **1‐*PP*** (solid lines) and **1‐*MM*** (dashed lines) and corresponding time‐dependent spectra after the addition of *rac*‐[5]‐helicene. Inset: CD spectra of [5]‐helicene after complexation with **1‐*PP*** (solid lines) and **1‐*MM*** (dashed lines) and subsequent GPC separation (complexation in chloroform: green; complexation in tetrachloromethane: purple) in chloroform at room temperature. For comparison, the corresponding CD spectrum of *rac*‐[5]‐helicene is also shown (black solid line). Furthermore, an illustration of the template‐controlled deracemization of *rac*‐[5]‐helicene is shown. b) Time‐dependent changes in the CD absorption of enantiopure *M*‐[5]‐helicene in the absence and presence of **1‐*PP*** recorded in tetrachloromethane at room temperature.

Therefore, solutions that had been equilibrated for 14 h were subjected to GPC separation of the host and guest to afford [5]‐helicene with 27 % *ee* (average of results obtained for the *P*‐ and *M*‐enrichment; Figure 4 a, inset). A comparison of the CD spectrum with that of enantiomerically pure [5]‐helicene^[34]^ (Figure S46a) shows that we obtained an excess of *M*‐[5]‐helicene with the **1‐*MM*** host, while an enrichment of *P*‐[5]‐helicene was observed after complexation with **1‐*PP***. If the same deracemization experiment was carried out in the less‐polar solvent tetrachloromethane, the *ee* value was increased to 66 % (Figure 4 a, inset).^[35]^ This observation shows that an increased binding affinity leads to a more efficient chirality transfer from the host to the guest and, thus, indicates that the complexation is responsible for the observed deracemization. Time‐dependent CD studies suggest that this interconversion between the enantiomeric forms of [5]‐helicene is catalyzed by the cyclophane template, with a significant acceleration of the enantiomerization process of *M*‐[5]‐helicene in the presence of **1‐*PP*** (Figures 4 b and S47).

In summary, we have reported the first inherently chiral perylene bisimide cyclophanes and their high binding affinity for carbohelicenes with association constants up to *K*_a_=3.9×10^10^ m ^−1^. Subsequently, we demonstrated chirality transfer from the cyclophane host to helicene guests by single‐crystal X‐ray analysis for the complex with [4]‐helicene as well as the cyclophane‐template‐catalyzed deracemization of [5]‐helicene.

## Conflict of interest

The authors declare no conflict of interest.

## Supporting information

As a service to our authors and readers, this journal provides supporting information supplied by the authors. Such materials are peer reviewed and may be re‐organized for online delivery, but are not copy‐edited or typeset. Technical support issues arising from supporting information (other than missing files) should be addressed to the authors.

Supplementary
