# Supplementary material for: Deracemization of Carbohelicenes by a Chiral Perylene Bisimide Cyclophane Template Catalyst
Source: Angew Chem Int Ed Engl. 2021 Jun 3;60(28):15323–7. doi: 10.1002/anie.202104591 (PMC8362091; doi:10.1002/anie.202104591)
Supplement: Supplementary file 1 — Supplementary [file ANIE-60-15323-s001.pdf]

Angewandte Chemie
Eine Zeitschrift der Gesellschaft Deutscher Chemiker

Supporting Information

Deracemization of Carbohelicenes by a Chiral Perylene Bisimide Cyclophane Template Catalyst

*Manuel Weh, Jessica Rühe, Benedikt Herbert, Ana-Maria Krause, and Frank Würthner**

anie_202104591_sm_miscellaneous_information.pdf

Table of Contents

General Methods S3

Synthetic Procedure S4

NMR Spectra S10

Mass Spectra S18

Separation of Diastereomers S22

Optical Spectroscopy S23

Titration Studies S25

DFT Calculations S33

2D NMR spectra S35

Single Crystal X-ray Analysis S37

Deracemization Experiments S39

References S42

General Methods

Chemicals: All chemicals and solvents were purchased from commercial suppliers and used without further purification. 1,7-dibromoperylene-3,4,9,10-tetracarboxy tetrabutylester, 3-(but-3-en-1-yloxy)phenol and (*Z*)-1,2-di(naphthalen-2-yl)ethene were synthesized according to literature known procedures.[S1-S4] *Rac*-[5]-helicene was synthesized in a similar procedure as reported in literature[S4] with (*Z*)-1,2-di(naphthalen-2-yl)ethene (109 mg, 389 µmol) and iodine (32 mg, 126 µmol), dissolved in cyclohexane and irradiated with UV/vis light (λ = 254–575 nm) for 1 h. The resolution of the enantiomers of [5]-helicene could be achieved by chiral HPLC (DCM/*n*-hexane 3:7, flow rate 6.5 mL/min). The enantiomeric excess was determined by analytical HPLC (Reprosil 100 Chiral-NR 8µm, Trentec, *n*-hexane/DCM 9:1) with values of *ee* > 98%. Subsequently, the CD spectra of the enantiomers were recorded (Figure S46a) and compared to the ones in literature in order to assign the two isomers.[S5] By rearrangement of equation S1 (see the part on “Deracemization Studies”), the $\Delta\varepsilon$ values of the enantiopure compound in chloroform were calculated and used for the calculation of the enantiomeric excess.

HPLC: Analytical HPLC was carried out on a JASCO system (PU 2080 PLUS) with a diode array detector (MD 2015), equipped with a ternary gradient unit (DG-2080-533) and inline-degasser (LG 2080-02). Recycling semipreparative HPLC was carried out on a JAI LC-9105. Chiral resolution was carried out using a Trentec Reprosil-100 Chiral-NR 8µm-column.

GPC: Gel permeation chromatography (GPC) was performed on a Shimadzu Recycling GPC-System (LC-20AD Prominence Pump; SPDMA20A Prominence Diode Array Detector) with three or two preparative columns (Japan Analytical Industries Co., Ltd.; JAIGEL-1 H, JAIGEL-2H and JAIGEL-2.5 H) in chloroform (HPLC grade, stabilized with 0.1 % EtOH) with a flow rate of 6.5 or 5.0 mL/min.

NMR spectroscopy: ^{1}H NMR and ^{13}C NMR spectra were recorded on a Bruker Avance III HD 400 spectrometer at 298 K. Chemical shift data are reported in parts per million (ppm, δ scale) downfield from tetramethylsilane and referenced internally to the residual proton (for proton NMR) in the solvent ($CDCl_3$: δ = 7.26; CD_2Cl_2: δ = 5.32; $C_2D_2Cl_4$ δ = 6.00) or to the carbon resonance ($CDCl_3$: δ = 77.16; CD_2Cl_2: δ = 53.84, $C_2D_2Cl_4$: δ = 74.20). The coupling constants are listed in Hertz.

Mass spectrometry: The MALDI-TOF mass spectra were measured with a Bruker Daltonics ultrafleXtreme mass spectrometer by using DCTB as a matrix. High-resolution ESI TOF spectra were acquired on a Bruker Daltonics micrOTOF focus spectrometer.

Melting points: Melting points were measured with an Olympus BX41 polarization microscope connected to a TP84 Linkam scientific temperature regulator.

Optical UV/vis absorption spectroscopy: All spectroscopic measurements were carried out under ambient conditions using solvents of spectroscopic grade. The absorption spectra were recorded on a JASCO V-770 or V-670 spectrometer equipped with a PAC-743R Peltier for temperature control.

Ciruclar dichroism (CD) spectroscopy: CD spectra were measured with a Jasco J-810 spectropolarimeter equipped with a JASCO CDF-426S Peltier temperature controller.

Steady-State fluorescence spectroscopy: Fluorescence spectra were recorded on an Edingburgh Instruments FLS981 fluorescence spectrometer. The quantum yields were determined by using the dilution method (*A* < 0.05) with *N,N`*-bis[2,6-diisopropylphenyl]perylene-3,4:9,10- bis(dicarboximide) (ϕ_F = 100% in chloroform)[S6] as a reference compound.

Single crystal X-ray analysis: The diffraction images for X-ray crystallographic analysis were collected on a Bruker D8 Quest Kappa diffractometer with a Photon II CPAD area detector using Cu Kα radiation.

Synthetic Procedure

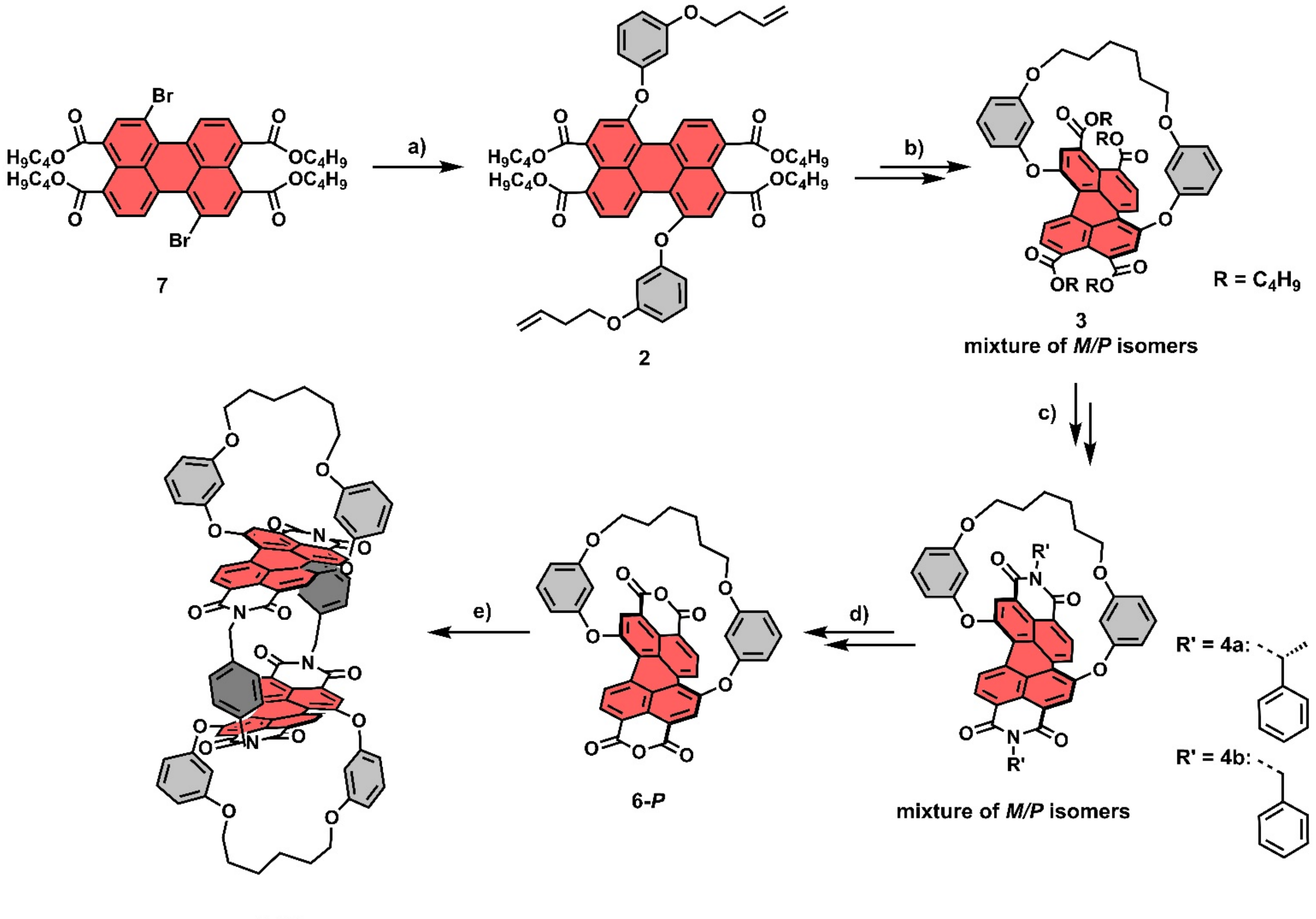

Scheme S1. Synthetic route to PBI cyclophane **1-*PP*** (route to **1-*MM*** analogous). In addition, the chemical structures of PBI dye **4b** is shown. Reaction conditions: a) 3-(but-3-en-1-yloxy)phenol, Cs_2CO_3, DMF, 100 °C, 7.5 h, 49%; b) 1. Grubbs II catalyst, DCM, reflux, 2 h, 82%; 2. H_2, Pd/C, EtOAc/MeOH (10:1), 2.5 h, 97%; c). 1. *p*-TsOH · H_2O, toluene, 140 °C, 17 h, quantitative, 2. **4a**: *R*-1-phenylethylamine, quinoline, $Zn(OAc)_2$, 140 °C, 5 h, 90% (diastereomeric mixture), d) 1. Chiral HPLC resolution of **4a**; 2. KOH, *tert*-butanol, 90 °C, 2 h, 72–90%; e) *para*-xylylenediamine, imidazole, toluene, 120 °C, 16 h, 17%.

1,7-Bis(3-(but-3-en-1-yloxy)phenoxy)-perylene-3,4,9,10-tetracarboxy tetrabutylester (2)

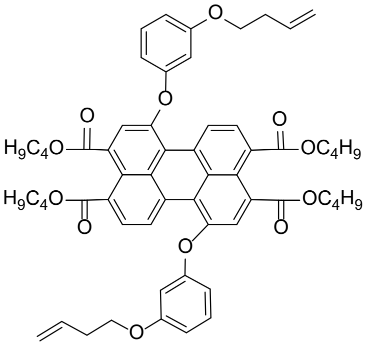

1,7-dibromoperylene-3,4,9,10-tetracarboxy tetrabutylester (**7**) (513 mg, 633 µmol), 3-(but-3-en-1-yloxy)phenol (408 mg, 2.48 mmol) and caesium carbonate (1.21 g, 3.73 mmol) were dissolved in dry DMF (21 mL) and stirred for 7.5 h at 100 °C under a nitrogen atmosphere. The mixture was cooled down to room temperature and DCM (100 mL) was added. Afterwards, the organic phase was washed with water (2 × 100 mL) and dried over Na_2SO_4. The solvent was removed under vacuum and the crude product purified by column chromatography (silica gel, DCM/cyclohexane 9:1 and then DCM/MeOH 99:1).

Yield: 302 mg (309 µmol, 49%) of a red, waxy solid.

^{1}H NMR (400 MHz, CD_2Cl_2): δ = 9.06 (d, ^3J = 8.1 Hz, 2H), 7.96 (d, ^3J = 8.1 Hz, 2H), 7.71 (s, 2H), 7.27–7.23 (m, 2H), 6.71 (ddd, ^3J = 8.5 Hz, ^4J = 2.3 Hz, ^4J = 0.9 Hz, 2H), 6.65–6.62 (m, 4H), 5.87 (ddt, ^3J = 17.3 Hz, ^3J = 10.2 Hz, ^3J = 6.8 Hz, 2H), 5.15–5.04 (m, 4 H), 4.24 (t, ^3J = 6.8 Hz, 4H), 4.19 (t, ^3J = 6.8 Hz, 4H), 3.97 (t, ^3J = 6.6 Hz, 4H), 2.50–2.48 (m, 4H), 1.73–1.63 (m, 8H), 1.45–1.33 (m, 8H), 0.95 (t, ^3J = 7.4 Hz, 6H), 0.89 (t, ^3J = 7.4 Hz, 6H).

^{13}C NMR (101 MHz, CD_2Cl_2): δ = 168.6, 167.8, 161.0, 157.3, 152.1, 134.9, 132.3, 131.9, 131.5, 131.0, 130.1, 129.5, 127.6, 125.2, 125.1, 123.2, 117.2, 111.0, 110.5, 105.8, 67.9, 65.8, 65.6, 33.9, 31.0, 30.8, 19.6, 19.5, 13.94, 13.90.

HRMS (ESI, positive, acetonitrile/chloroform): (*m/z*) [*M*+Na]$^+$, calcd. for $C_{60}H_{64}NaO_{12}^+$: 999.4290; found: 999.4289.

m.p.: 44–46 °C.

***rac-(E/Z)*-1,7-(3,3'-(hex-3-en-1,6-diylbis(oxy))diphenolate)-perylene 3,4,9,10-tetracarboxy tetrabutylester (5)**

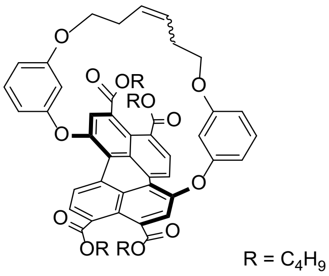

R = C_4H_9

mixture of ***M/P*** and ***E/Z*** isomers

A solution of 2nd Generation Grubbs Catalyst (1,3-bis(2,4,6-trimethylphenyl)-2-imid-azolidinylidene)dichloro(phenylmethylene)-(tricyclohexylphosphine)ruthenium) (20.4 mg, 24.0 µmol, 9 mol%) in dry DCM (90 mL) was added to a mixture of 1,7-bis(3-(but-3-en-1-yloxy)phenoxy perylene-3,4:9,10-tetracarboxy tetrabutylester (**2**) (263.0 mg, 0.277 mmol) in dry DCM (40 mL). The resulting solution was stirred under a nitrogen atmosphere under reflux for 2 h. The solvent was evaporated under reduced pressure and the crude product was purified by column choromatography (silica gel, DCM).

Yield: 216 mg (228 µmol, 82%) of a yellow solid.

^{1}H NMR (400 MHz, CD_2Cl_2): δ = 9.06 (d, ^3J = 8.1 Hz, 2H), [8.99 (d, ^3J = 8.0 Hz, 0.17 H)], 7.97 (d, ^3J = 8.1 Hz, 2H), [7.92 (d, ^3J = 8.0 Hz, 0.18 H)], [7.81 (s, 0.17H)], 7.77 (s, 2H), 7.29 (t, ^3J = 8.2 Hz, 2H), 7.01 (ddd, ^3J = 8.2 Hz, ^4J = 2.3 Hz, ^4J = 0.8 Hz, 2H), 6.51 (ddd, ^3J = 8.2 Hz, ^4J = 2.3 Hz, ^4J = 0.8 Hz, 2H), 5.79 (t, ^4J = 2.3 Hz, 2H), 5.36–5.34 (m, 2H), 4.20–4.32 (m, 8H), 3.64 (t, ^3J = 6.3 Hz, 4H), 2.29–2.24 (m, 4H), 1.78–1.68 (m, 8H), 1.50–1.38 (m, 8H), 0.99–0.93 (m, 12H). [main isomer; *main:minor* ratio: *12*/*1*] Additional peaks with significant lower intensity are observed resulting from the presence of different isomers.

^{13}C NMR (101 MHz, CD_2Cl_2): δ = 168.6, 167.8, 160.7, 158.4, 151.1, 132.43, 132.36, 131.1, 130.7, 130.4, 129.8, 128.8, 127.8, 127.7, 125.9, 125.2, 111.2, 108.4, 102.5, 67.4, 66.0, 65.8, 32.6, 31.0, 30.9, 19.63, 19.58, 13.95, 13.93.

HRMS (ESI, positive, acetonitrile/chloroform): (*m/z*) [*M*+Na]$^+$, calcd. for $C_{58}H_{60}NaO_{12}^+$: 971.3977; found: 971.3963.

m.p.: 160–163 °C.

***rac*-1,7-(3,3'-(hexane-1,6-diylbis(oxy))diphenolate)-perylene-3,4,9,10-tetracarboxy tetrabutylester (3)**

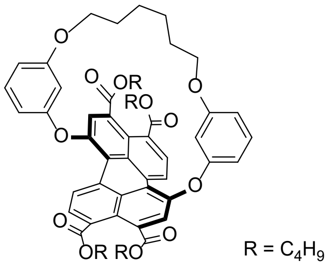

R = C_4H_9

mixture of ***M/P*** isomers

Compound **5** (188 mg, 198 µmol) was dissolved in a solvent mixture of ethyl acetate and methanol (10:1, v/v, 130 mL) and then degassed for 15 min by bubbling argon through the solution. Afterwards, Pd/C (80 mg, 10% Pd) was added, the solution was again degassed for 15 min and finally stirred at room temperature for 2.5 h under a hydrogen atmosphere (atmospheric pressure). The catalyst was removed by filtration over celite and the solvent was removed under reduced pressure.

Yield: 183 mg (192 µmol, 97%) of a yellow solid.

^{1}H NMR (400 MHz, CD_2Cl_2): δ = 9.05 (d, ^3J = 8.1 Hz, 2H), 7.97 (d, ^3J = 8.1 Hz, 2H), 7.77 (s, 2H), 7.28 (t, ^3J = 8.2 Hz, 2H), 7.00 (ddd, ^3J = 8.2 Hz, ^4J = 2.3 Hz, ^4J = 0.9 Hz, 2H), 6.51 (ddd, ^3J = 8.3 Hz, ^4J = 2.3 Hz, ^4J = 0.9 Hz, 2H), 5.80 (t, ^4J = 2.3 Hz, 2H), 4.32–4.20 (m, 8H), 3.66–3.56 (m, 4H), 1.78–1.69 (m, 8H), 1.60–1.55 (m, 4H), 1.50–1.20 (m, 12H), 1.00–0.93 (m, 12H).

^{13}C NMR (101 MHz, CD_2Cl_2): δ = 168.6, 167.9, 160.9, 158.3, 151.3, 132.4, 132.3, 131.1, 130.6, 130.3, 129.7, 127.8, 127.5, 125.8, 125.0, 111.2, 108.7, 102.6, 68.0, 66.0, 65.8, 31.0, 30.9, 29.4, 26.3, 19.63, 19.58, 13.95, 13.93.

HRMS (ESI, positive, acetonitrile/chloroform): (*m/z*) [*M*+Na]$^+$, calcd. for $C_{58}H_{62}NaO_{12}^+$: 973.4134; found: 973.4111.

m.p.: 151–154 °C.

***rac*-1,7-(3,3'-(hexan-1,6-diylbis(oxy))diphenolate)-perylene-3,4:9,10-tetracarboxylic bisanhydride (6)**

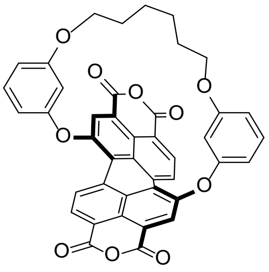

mixture of ***M/P*** isomers

Compound **3** (39.9 mg, 42.0 µmol) and *p*-TsOH · H_2O (89.4 mg, 470 µmol) were dissolved in toluene (7 mL) and stirred for 19 h at 100 °C. After cooling the mixture down to room temperature, the solvent was removed under reduced pressure. Water (10 mL) was added and the mixture sonicated for 5 min. The supernatant water was removed by centrifugation. The washing procedure was repeated twice to obtain a red solid, which was used without further purification.

Yield: 29.0 mg (42.0 µmol, quant.) of a red solid.

^{1}H NMR (400 MHz, $CDCl_3$): δ = 9.43 (d, ^3J = 8.2 Hz, 2H), 8.64 (d, ^3J = 8.2 Hz, 2H), 8.46 (s, 2H), 7.35 (t, ^3J = 8.3 Hz, 2H), 7.08 (ddd, ^3J = 8.3 Hz, ^4J = 2.3 Hz, ^4J = 0.9 Hz, 2H), 6.55 (ddd, ^3J = 8.3 Hz, ^4J = 2.3 Hz, ^4J = 0.9 Hz, 2H), 5.58 (t, ^4J = 2.3 Hz, 2H), 3.61–3.49 (m, 4H), 1.61–1.55 (m, 4H), 1.26–1.19 (m, 4H).

MS (MALDI-Tof, positive, DCTB 1:3 in chloroform): (*m/z*) [*M*]$^+$, calcd. for $C_{42}H_{26}O_{10}^+$: 690.15260; found: 690.15214.

m.p.: 200 °C.

***(M/P)-R-N,N'*-(1-phenylethylamine)-1,7-(3,3'-(hexan-1,6-diylbis(oxy))diphenolate)-perylene-3,4:9,10-tetracarboxylic acid bisimide (4a)**

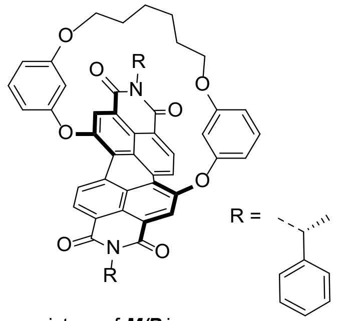

mixture of ***M/P*** isomers

Racemic perylene bisanhydride **6** (164 mg, 237 µmol), *R*-phenylethylamine (1.50 mL, 1.44 g, 11.9 mmol) and $Zn(OAc)_2$ (117 mg, 638 µmol) were mixed together in freshly distilled quinoline (4.5 mL) and stirred for 5 h at 140 °C under a nitrogen atmosphere. After being cooled down to room temperature, the reaction mixture was diluted with DCM (50 mL) and washed with 1 M HCl (4 × 60 mL). The organic layer was dried over Na_2SO_4 and the solvent removed under reduced pressure. The crude product was purified by column chromatography (silica gel, DCM). The product was dissolved in a minimum amount of chloroform and precipitated by the addition of methanol. The precipitate was washed three times with *n*-hexane to give a red solid. The resulting diastereomers were separated by chiral HPLC (Reprosil, DCM:*n*-hexane 55:45, flow rate: 6.5 mL/min , see figure S28c)).

Isomeric mixture:

Yield: 194 mg (216 µmol, 90%) of a red solid (isomeric mixture).

HRMS (ESI, positive, acetonitrile/chloroform): (*m/z*) $[M+Na]^+$, calcd. for $C_{58}H_{44}N_2NaO_8^+$: 919.29899; found: 919.29712.

Isomer 4a-*P*: Retention time (Trentec, Reprosil 100 chiral-NR, Ø = 0.8 cm, DCM/*n*-hexane (55:45), flow: 1.0 mL/min): 12.4 min; λ_{max} ($\Delta\varepsilon$): 528 nm (+ 46 M^{-1} cm $^{-1}$). **4a-*M***: Retention time (Trentec, Reprosil 100 chiral-NR, Ø = 0.8 cm, DCM/*n*-hexane (55:45), flow: 1.0 mL/min): 13.6 min; λ_{max} ($\Delta\varepsilon$): 528 nm (– 48 M^{-1} cm^{-1}). The stereochemical assignment of the isolated diastereomers was achieved by comparison of the CD spectra with those of the previously reported, structurally similar, epimerically pure *P*- and *M*- configured macrocyclic PBIs.[S7]

Isomer 4a-*P*:

^{1}H NMR (400 MHz, CD_2Cl_2): δ = 9.37 (d, ^3J = 8.3 Hz, 2H), 8.52 (d, ^3J = 8.3 Hz, 2H), 8.37 (s, 2H), 7.46–7.44 (m, 4H), 7.32–7.27 (m, 6H), 7.24–7.22 (m, 2H), 7.06 (ddd, ^3J = 8.2 Hz, ^4J = 2.5 Hz, ^4J = 0.9 Hz, 2H), 6.50–6.47 (m, 4H), 5.66 (t, ^4J = 2.4 Hz, 2H), 3.57–3.50 (m, 4H), 1.95 (d, ^3J = 7.1 Hz, 6H), 1.56–1.5 (m, 4H), 1.21–1.16 (m, 4H).

^{13}C NMR (101 MHz, CD_2Cl_2): δ = 163.2, 162.7, 160.5, 157.8, 153.3, 140.8, 132.5, 130.66, 130.57, 129.3, 129.0, 128.4, 128.1, 127.0, 126.9, 126.7, 126.4, 125.1, 123.1, 111.0, 108.6, 101.6, 67.6, 50.3, 28.9, 26.0, 16.0.

m.p.: 216–218 °C.

UV/vis ($CHCl_3$, c_T = 10 µM, nm): λ_{max} (ε_{max}, M^{-1} cm^{-1}) = 528 (51000).

Fluorescence ($CHCl_3$, nm): λ_{max} (λ_{ex}) = 548 (490).

CD ($CHCl_3$, nm): λ_{max} ($\Delta\varepsilon_{max}$, M^{-1} cm^{-1}): 528 (47).

Isomer 4a-*M*:

^{1}H NMR (400 MHz, CD_2Cl_2): δ = 9.31 (d, ^3J = 8.3 Hz, 2H), 8.52 (d, ^3J = 8.3 Hz, 2H), 8.27 (s, 2H), 7.45–7.43 (m, 4H), 7.30–7.26 (m, 6H), 7.21–7.19 (m, 2H), 7.03 (ddd, ^3J = 8.2 Hz, ^4J = 2.5 Hz, ^4J = 0.9 Hz, 2H), 6.50–6.44 (m, 4H), 5.57 (t, ^3J = 2.4 Hz, 2H), 3.51–3.44 (m, 4H), 1.94 (d, ^3J = 7.1 Hz, 6H), 1.50–1.48 (m, 4H), 1.14–1.10 (m, 4H).

^{13}C NMR (101 MHz, CD_2Cl_2): δ = 163.3, 162.5, 160.4, 157.7, 153.2, 140.8, 132.4, 130.7, 129.1, 128.9, 128.3, 128.1, 127.1, 126.9, 126.6, 126.3, 125.1, 123.0, 111.0, 108.6, 101.5, 67.6, 50.3, 28.9, 25.9, 16.0.

m.p.: 204–207 °C.

UV/vis ($CHCl_3$, c_T = 10 µM, nm): λ_{max} (ε_{max} M^{-1} cm^{-1}) = 528 (51000).

Fluorescence ($CHCl_3$, nm): λ_{max} ($\lambda_{ex,\,nm}$) = 548 (490).

CD ($CHCl_3$, nm): λ_{max} ($\Delta\varepsilon_{max}$, M^{-1} cm^{-1}): 528 (–48).

(*P/M*)-1,7-(3,3'-(hexane-1,6-diylbis(oxy))diphenolate) perylene-3,4:9,10-tetracarboxylic bisanhydride (6-*P*/6-*M*)

6-*P*:

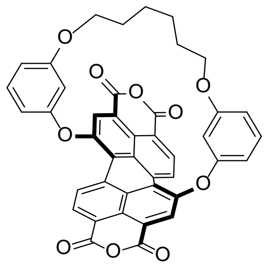

Perylenbisimide **4a-*P*** (152 mg, 169 µmol) was stirred together with KOH (488 mg, 8.70 mmol) in *tert*-butanol (32 mL) under a nitrogen atmosphere for 2 h at 90 °C. The reaction mixture was cooled down to room temperature. Subsequently, 1 M HCl (32 mL) was added to the mixture and it was stirred for 15 min. The supernatant solution was removed after centrifuging.
Yield: 83.5 mg (121 µmol, 72%) of a red solid.
^{1}H NMR (400 MHz, $CDCl_3$): δ = 9.43 (d, ^3J = 8.2 Hz, 2H), 8.64 (d, ^3J = 8.2 Hz, 2H), 8.46 (s, 2H), 7.35 (t, ^3J = 8.3 Hz, 2H), 7.08 (ddd, ^3J = 8.3 Hz, ^4J = 2.3 Hz, ^4J = 0.9 Hz, 2H), 6.55 (ddd, ^3J = 8.3 Hz, ^4J = 2.3 Hz, ^4J = 0.9 Hz, 2H), 5.58 (t, ^4J = 2.3 Hz, 2H), 3.61–3.49 (m, 4H), 1.61–1.55 (m, 4H), 1.26–1.19 (m, 4H).
^{1}H NMR is in accordance with the spectral data obtained from the racemic compound **6**.
m.p.: 201 °C.

6-*M*:

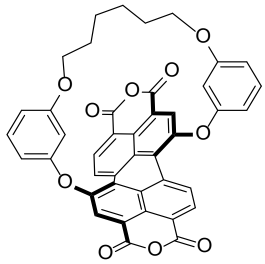

The synthesis was carried out analogue to the synthesis of **6-*P*** with perylenbisimide **4a-*M*** (118 mg, 132 µmol) and KOH (386 mg, 6.88 mmol) in *tert*-butanol (25 mL).
Yield: 81.8 mg (118 µmol, 90%) of a red solid.
^{1}H NMR (400 MHz, $CDCl_3$): δ = 9.43 (d, ^3J = 8.2 Hz, 2H), 8.64 (d, ^3J = 8.2 Hz, 2H), 8.46 (s, 2H), 7.35 (t, ^3J = 8.3 Hz, 2H), 7.08 (ddd, ^3J = 8.3 Hz, ^4J = 2.3 Hz, ^4J = 0.9 Hz, 2H), 6.55 (ddd, ^3J = 8.3 Hz, ^4J = 2.3 Hz, ^4J = 0.9 Hz, 2H), 5.58 (t, ^4J = 2.3 Hz, 2H), 3.61–3.49 (m, 4H), 1.61–1.55 (m, 4H), 1.26–1.19 (m, 4H).
^{1}H NMR is in accordance with the spectral data obtained from the racemic compound **6**.
m.p.: 200 °C.

***rac-N,N'*-benzylamine-1,7-(3,3'-(hexan-1,6-diylbis(oxy))diphenolate)-perylene-3,4:9,10-tetracarboxylic acid bisimide (4b)**

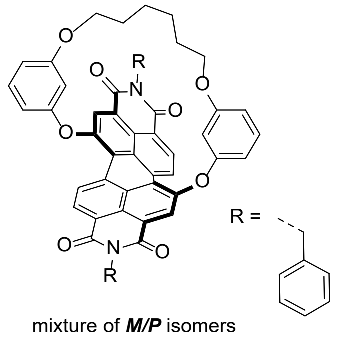

mixture of ***M/P*** isomers

Racemic perylene bisanhydride **6** (29.0 mg, 42.0 µmol), benzylamine (500 µL, 492 mg, 4.59 mmol) and $Zn(OAc)_2$ (27.0 mg, 147 µmol) were dissolved in freshly distilled quinolone (1.5 mL). The solution was stirred for 5.5 h at 140 °C under a nitrogen atmosphere. Afterwards, the reaction was cooled down to room temperature and DCM (80 mL) was added to the mixture, which was subsequently washed with 1 M HCl (2 × 100 mL). The organic layer was dried over Na_2SO_4 and the solvent removed under reduced pressure. The

red solid was dissolved in a minimum amount of DCM and was precipitated upon the addition of methanol. The precipitate was separated from the supernatant by centrifuging the mixture and further purified by GPC ($CHCl_3$, flow rate: 6.4 mL min^{-1}).
Yield: 13.0 mg (15.0 µmol, 36%) of a red solid.
^{1}H NMR (400 MHz, TCE-d_2): δ = 9.35 (d, ^3J = 8.2 Hz, 2H), 8.60 (d, ^3J = 8.2 Hz, 2H), 8.42 (s, 2H), 7.54–7.52 (m, 4H), 7.35–7.28 (m, 8H), 7.08 (dd, ^3J = 8.3 Hz, ^4J = 2.2 Hz, 2H), 6.51 (dd, ^3J = 8.3 Hz, ^4J = 2.2 Hz, 2H), 5.59 (t, ^3J = 8.3 Hz, 2 H), 5.34 (s, 4H), 3.55–3.47 (m, 4H), 1.55–1.47 (m, 4H), 1.18–1.12 (m, 4H).
^{13}C NMR (101 MHz, TCE-d_2): δ = 163.6, 163.0, 160.4, 157.8, 153.5, 137.0, 132.7, 131.3, 131.2, 129.4, 129.2, 129.1, 128.9, 128.0, 127.1, 126.3, 124.7, 122.8, 111.4, 109.3, 101.7, 67.9, 44.0, 29.0, 26.3.
HRMS (ESI, positive, acetonitrile/chloroform): (*m/z*) [*M*+Na]$^+$, calc. for $C_{56}H_{40}N_2NaO_8^+$: 891.26769; found: 891.26888.
m.p.: >300 °C.
UV/vis ($CHCl_3$, c_T = 10 µM, nm): λ_{max} (ε_{max}, M^{-1} cm^{-1}) = 530 (55000).
Fluorescence ($CHCl_3$, nm): λ_{max} (λ_{ex}) = 550 (490).

Cyclophane 1-*PP*:

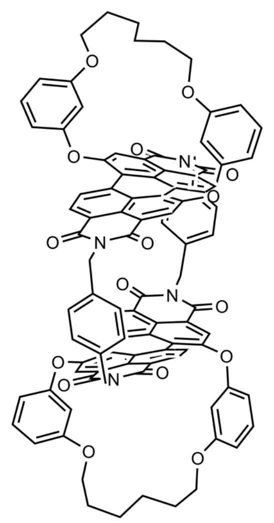

A mixture of perylene bisanhydride **6-*P*** (82.5 mg, 119 µmol), *para*-xylylenediamine (16.3 mg, 120 µmol) and imidazole (820 mg, 12.0 mmol) were stirred in toluene (200 mL) at 120 °C under a nitrogen atmosphere for 16 h. After cooling down to room temperature, the reaction mixture was washed with 1 M HCl (3 x 50 mL). The combined organic layers were dried over Na_2SO_4 and the organic solvent was removed under reduced pressure. The crude product was purified by column chromatography (chloroform/MeOH, 95.5:0.5) and GPC ($CHCl_3$, flow rate: 6.4 mL/min). The red solid was subsequently dissolved in a minimum amount of chloroform and precipitated by the addition of methanol. The suspension was centrifuged, the supernatant removed and the solid washed with *n*-hexane.
Yield: 15.9 mg (10.1 µmol, 17%) of a red solid.
^{1}H NMR (400 MHz, TCE-d_2): δ = 9.05–9.04 (br, 4H), 8.38–8.35 (m, 8H), 7.49 (s, 8H), 7.31 (t, ^3J = 8.1 Hz, 4H), 7.03 (dd, ^3J = 8.4 Hz, ^4J = 1.9 Hz, 4H), 6.52 (dd, ^3J = 8.4 Hz, ^4J = 1.9 Hz, 4H), 5.68 (t, ^3J = 2.1 Hz, 4H), 5.40 (d, ^3J = 13.2 Hz, 4H), 5.22 (d, ^3J = 13.3 Hz, 4H), 3.58–3.50 (m, 8H), 1.58–1.46 (m, 8H), 1.23–1.13 (m, 8H).
^{13}C NMR: see enantiomeric form (*vide infra*).
HRMS (ESI, positive, acetonitrile/chloroform): (*m/z*) [*M*]$^+$, calc. for $C_{100}H_{68}N_4O_{16}^+$: 1581.46639; found: 1581.47067.
m.p.: >300 °C.
UV/vis ($CHCl_3$, c_T = 10 µM, nm): λ_{max} (ε_{max}, M^{-1} cm^{-1}) = 528 (75000).
Fluorescence ($CHCl_3$, nm): λ_{max} (λ_{ex}) = 561 (490).
CD ($CHCl_3$, nm): λ_{max} ($\Delta\varepsilon_{max}$, M^{-1} cm^{-1}): 530 (+94).

Cyclophane 1-*MM*:

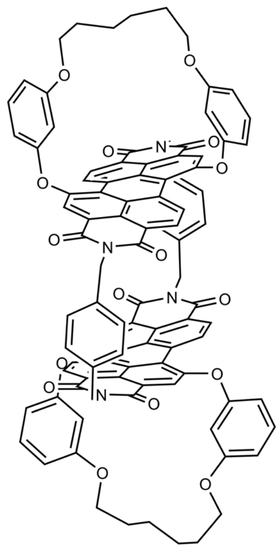

The synthesis was carried out analogue to the synthesis of **1-*PP*** with perylene bisanhydride **6-*M*** (81.4 mg, 118 µmol, (1eq.)), *para*-xylylenediamine (16.1 mg, 118 µmol, (1 eq.)) and imidazole (820 mg, 12.0 mmol) in toluene (200 mL).
Yield: 16.0 mg (10.1 µmol, 17%) of a red solid.
^{1}H NMR (400 MHz, TCE-d_2): δ = 9.04–9.03 (br, 4H), 8.38–8.35 (m, 8H), 7.49 (s, 8H), 7.31 (t, ^3J = 8.1 Hz, 4H), 7.03 (dd, ^3J = 8.4 Hz, ^4J = 1.9 Hz 4H), 6.52 (dd, ^3J = 8.4 Hz, ^4J = 1.9 Hz 4H), 5.68 (t, ^3J = 2.1 Hz, 4H), 5.40 (d, ^3J = 13.7 Hz, 4H), 5.22 (d, ^3J = 13.3 Hz, 4H), 3.60–3.48 (m, 8H), 1.60–1.44 (m, 8H), 1.23–1.13 (m, 8H).
^{13}C NMR (101 MHz, TCE-d_2): δ = 162.8, 162.4, 160.5, 157.6, 153.7, 137.3, 132.4, 131.2, 130.7, 130.6, 129.2, 128.5, 128.3, 126.3, 125.7, 124.6, 122.6, 111.6, 109.5, 101.9, 68.0, 42.8, 29.0, 26.3.
HRMS (ESI, positive, acetonitrile/chloroform): (*m/z*) [*M*+Na]$^+$, calc. for $C_{100}H_{68}N_4NaO_{16}^+$: 1603.45225; found: 1603.45260.
m.p.: >300 °C.
UV/vis ($CHCl_3$, c_T = 10 µM, nm): λ_{max} (ε_{max}, M^{-1} cm^{-1}) = 528 (75000).
Fluorescence ($CHCl_3$, nm): λ_{max} (λ_{ex}) = 561 (490). ϕ_{fl} ($CHCl_3$) = 0.2
CD ($CHCl_3$, nm): λ_{max} ($\Delta\varepsilon_{max}$, M^{-1} cm^{-1}): 530 (–89).

NMR Spectra

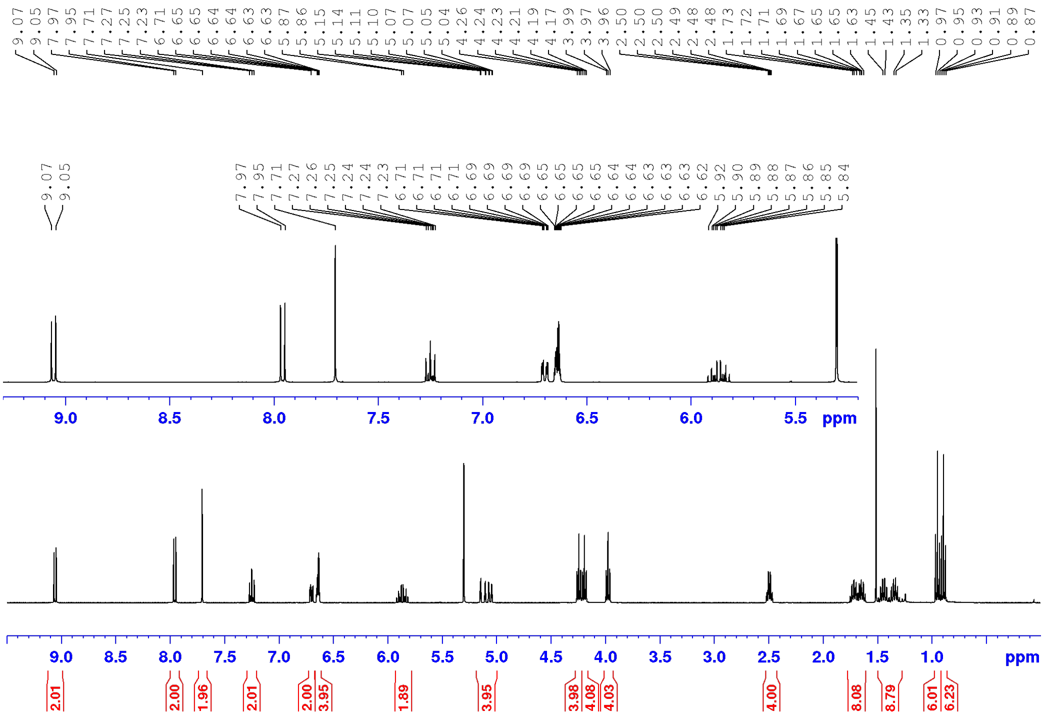

Figure S1. ^{1}H NMR (400 MHz) spectrum of compound **2** in CD_2Cl_2 at 298 K.

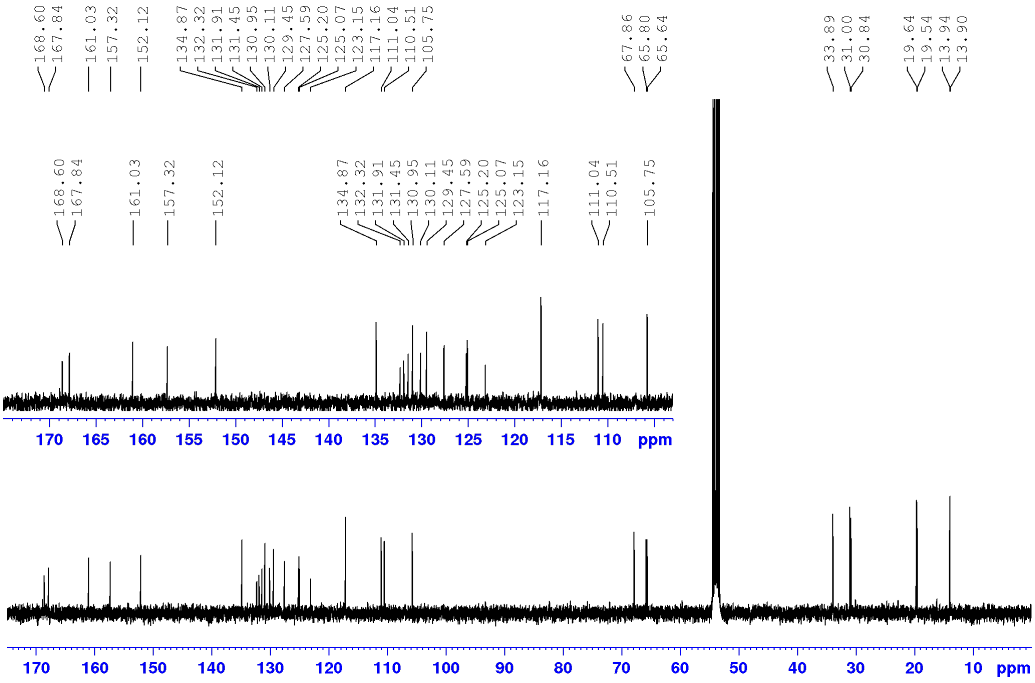

Figure S2. ^{13}C NMR (101 MHz) spectrum of compound **2** in CD_2Cl_2 at 298 K

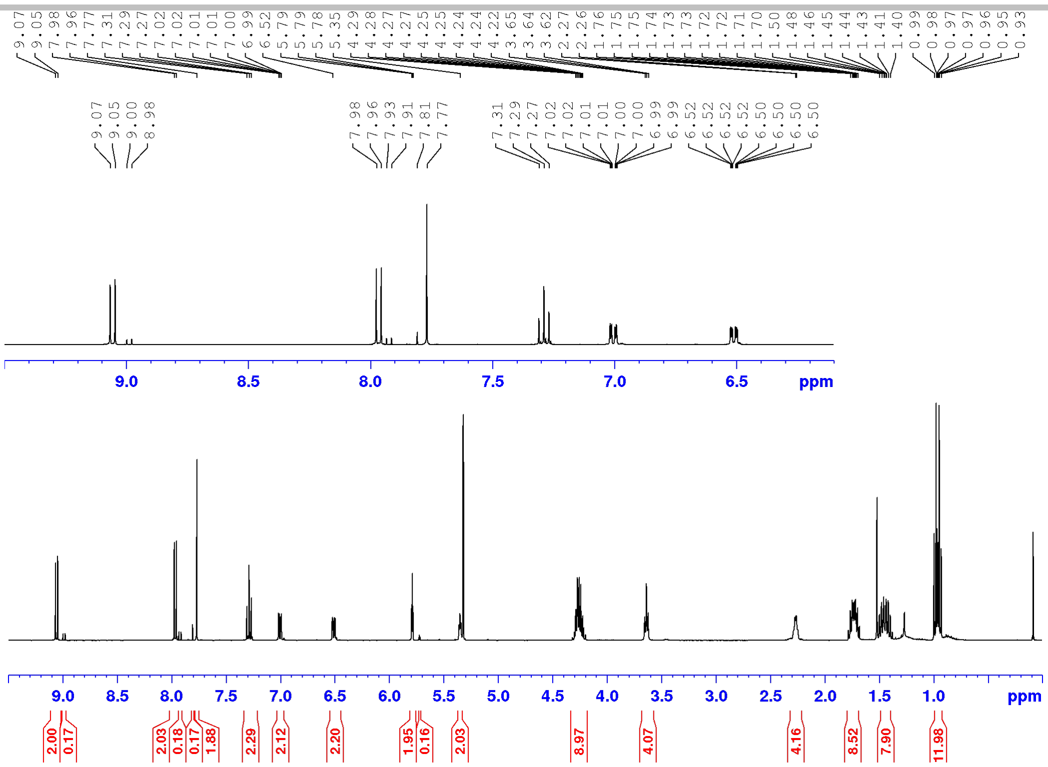

Figure S3. ^{1}H NMR (400 MHz) spectrum of compound **5** in CD_2Cl_2 at 298 K.

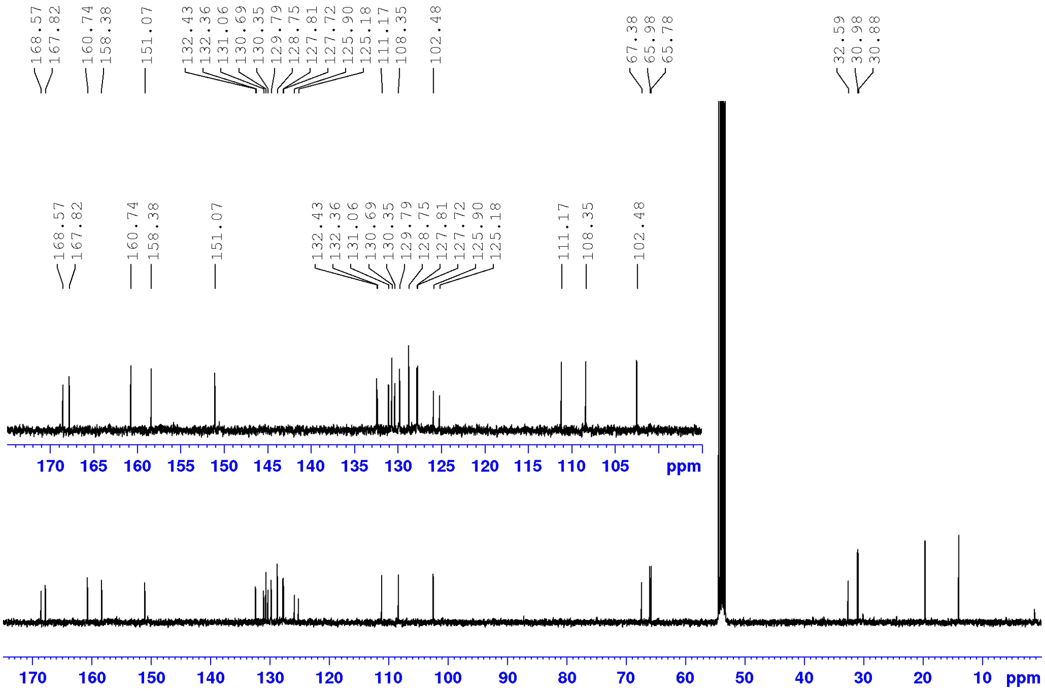

Figure S4. ^{13}C NMR (101 MHz) spectrum of compound **5** in CD_2Cl_2 at 298 K.

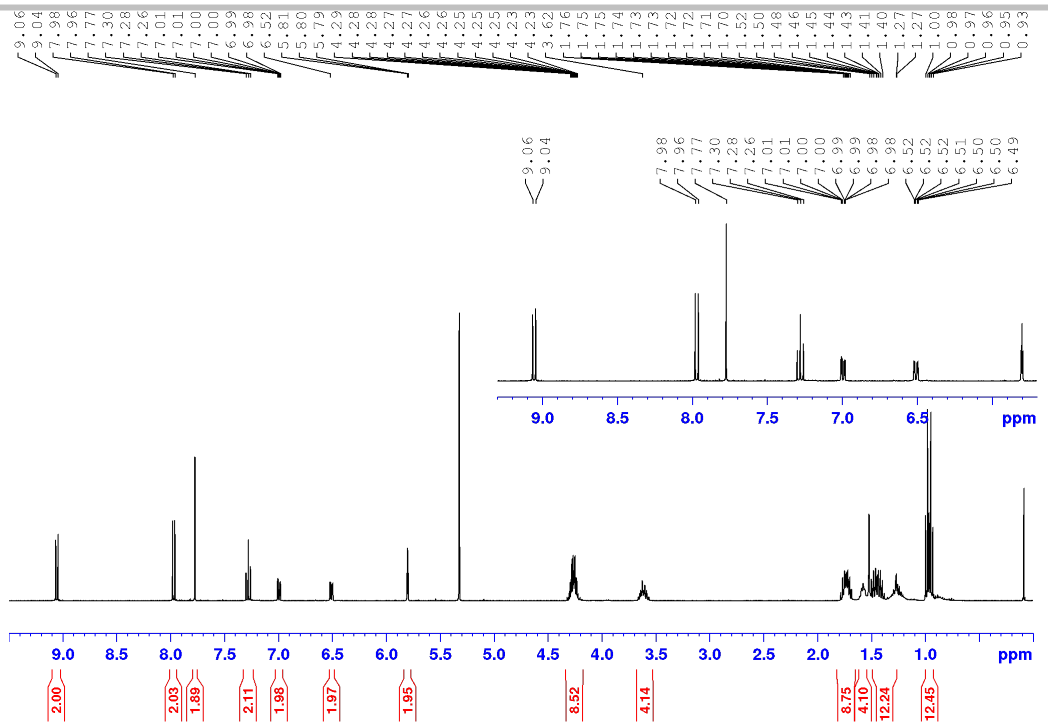

Figure S5. ^{1}H NMR (400 MHz) spectrum of compound **3** in CD_2Cl_2 at 298 K.

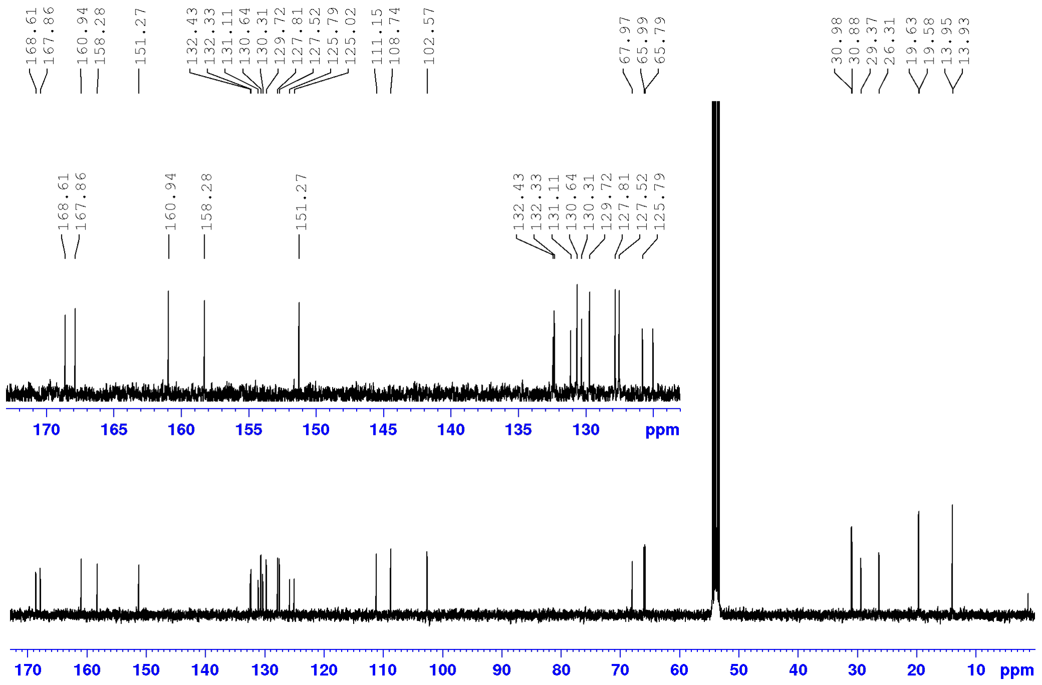

Figure S6. ^{13}C NMR (101 MHz) spectrum of compound **3** in CD_2Cl_2 at 298 K.

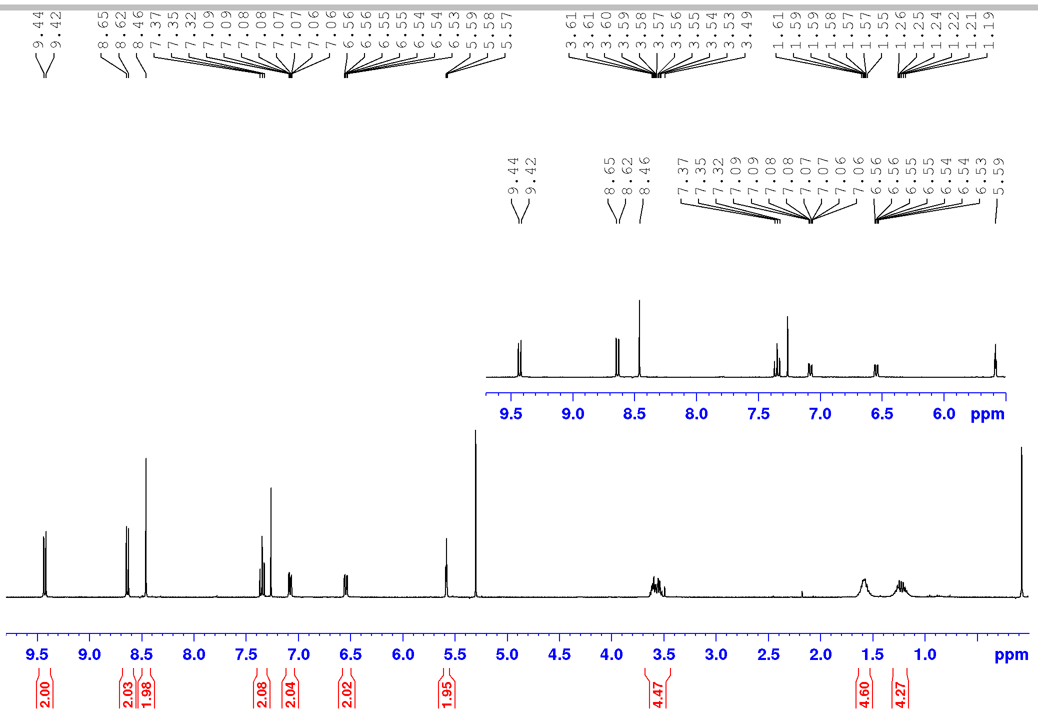

Figure S7. ^{1}H NMR (400 MHz) spectrum of compound **6** in CD_2Cl_2 at 298 K.

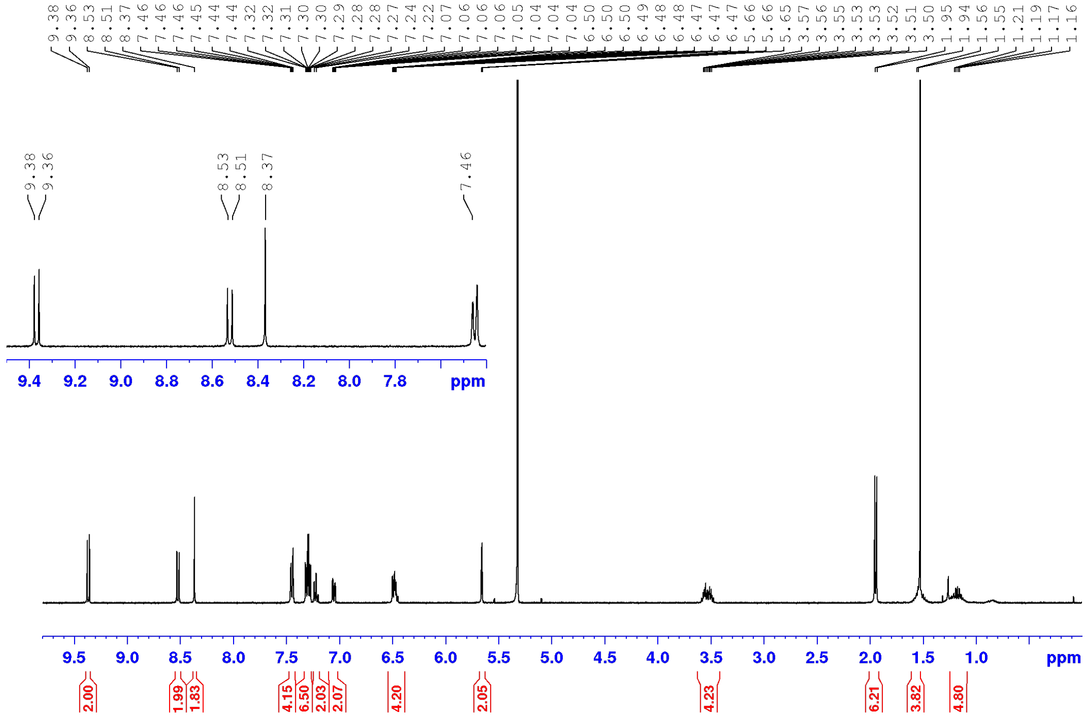

Figure S8. ^{1}H NMR (400 MHz) spectrum of compound **4a-*P*** in CD_2Cl_2 at 298 K.

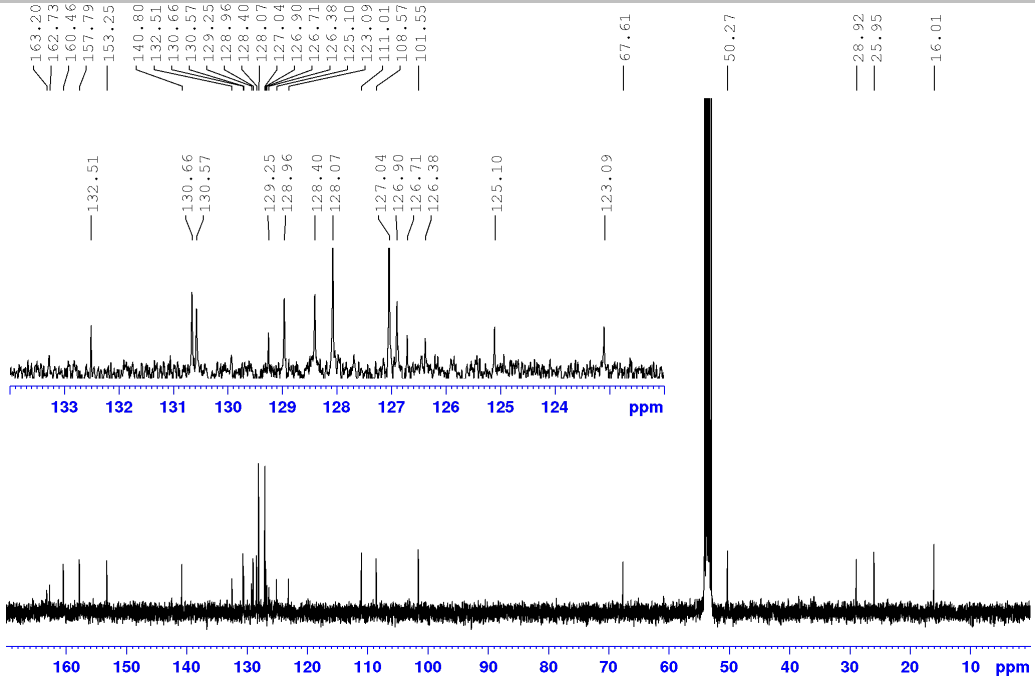

Figure S9. ^{13}C NMR (101 MHz) spectrum of compound **4a-*P*** in CD_2Cl_2 at 298 K.

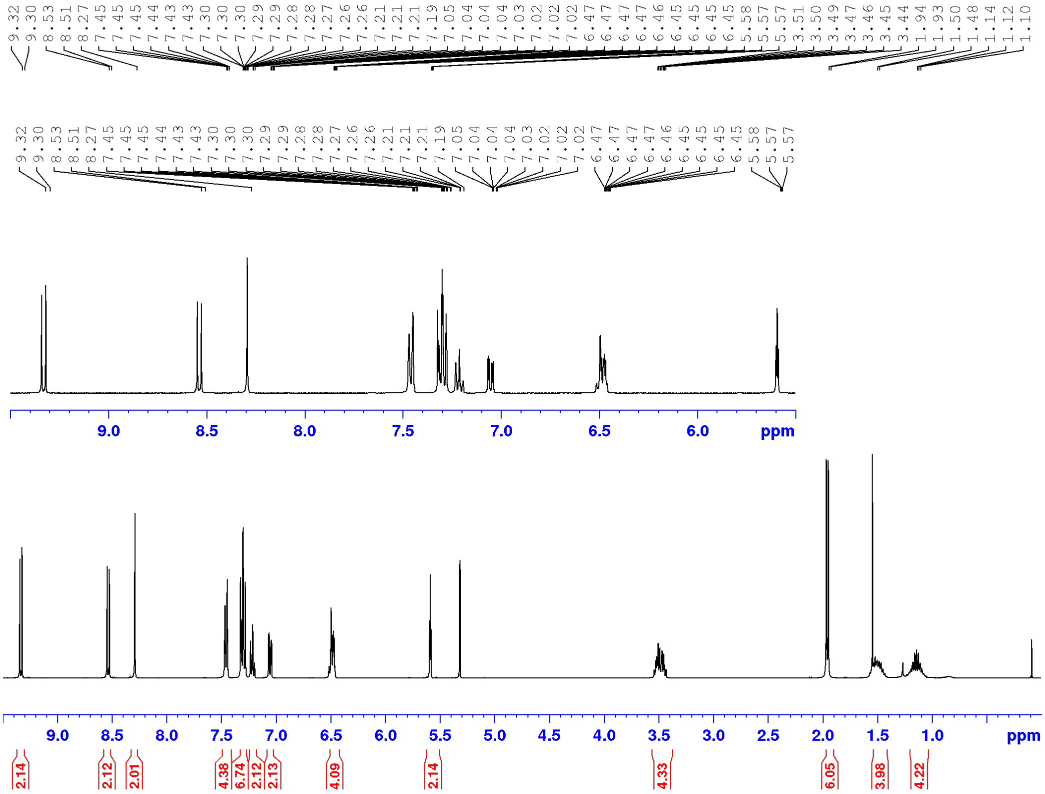

Figure S10. ^{1}H NMR (400 MHz) spectrum of compound **4a-*M*** in CD_2Cl_2 at 298 K.

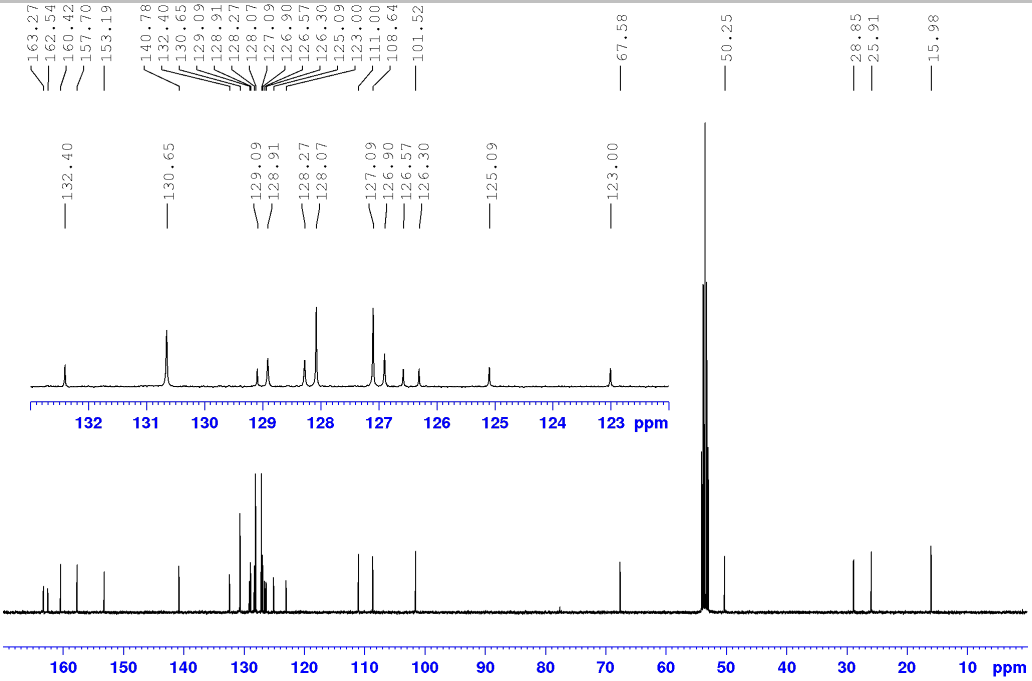

Figure S11. ^{13}C NMR (101 MHz) spectrum of compound **4a-*M*** in CD_2Cl_2 at 298 K.

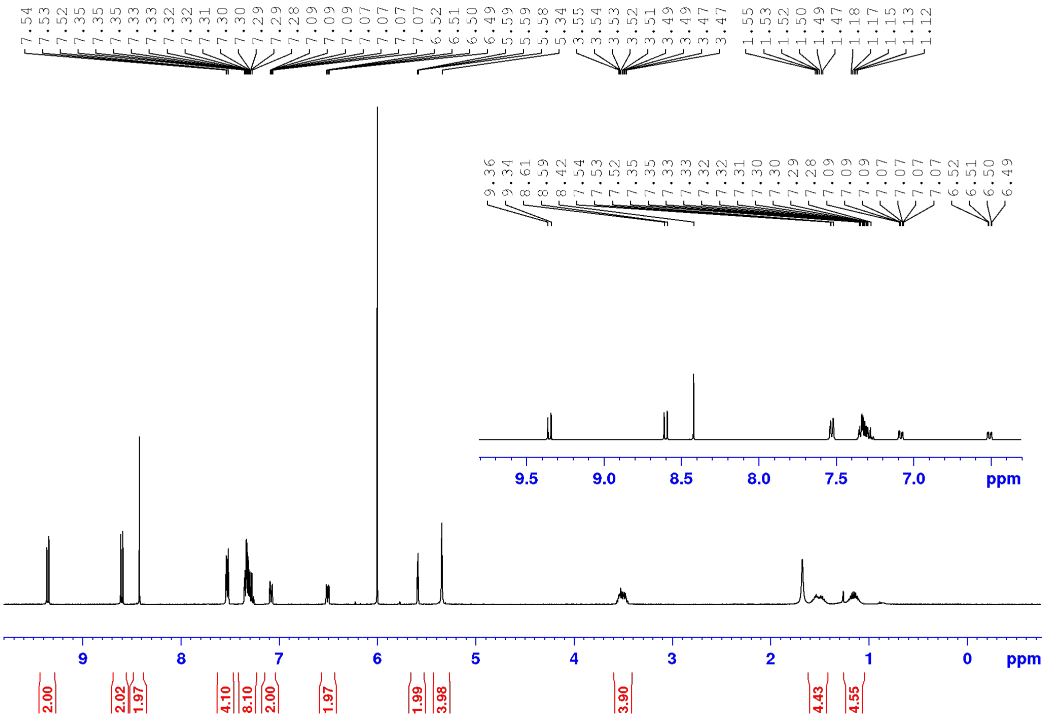

Figure S12. ^{1}H NMR (400 MHz) spectrum of compound **4b** in TCE-d_2 at 298 K.

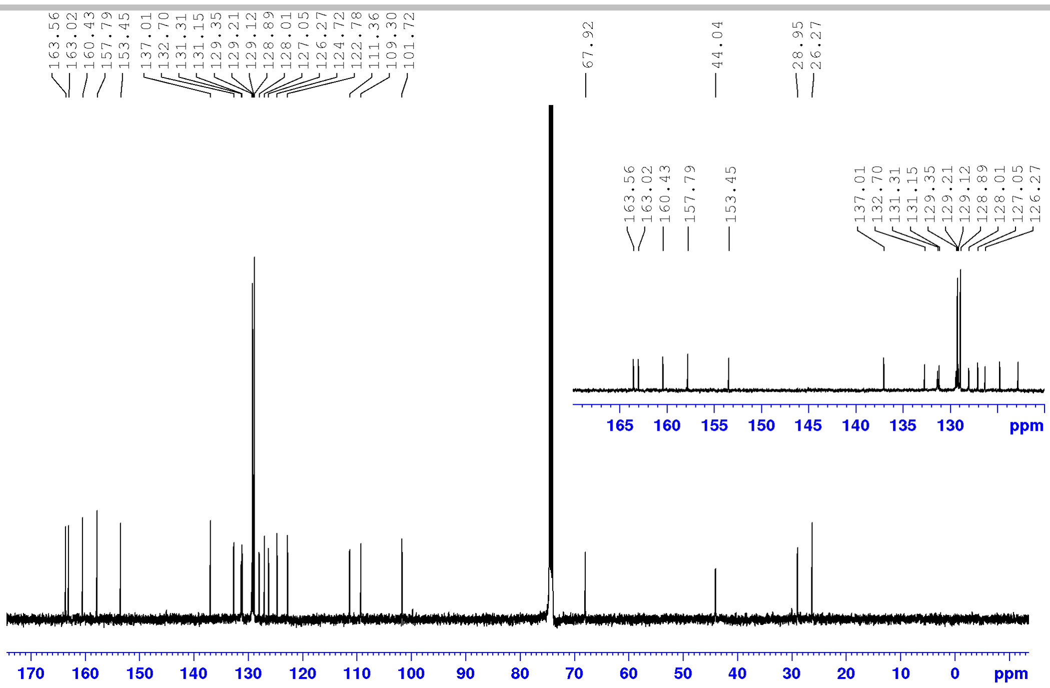

Figure S13. ^{13}C NMR (101 MHz) spectrum of compound **4b** in TCE-d_2 at 298 K.

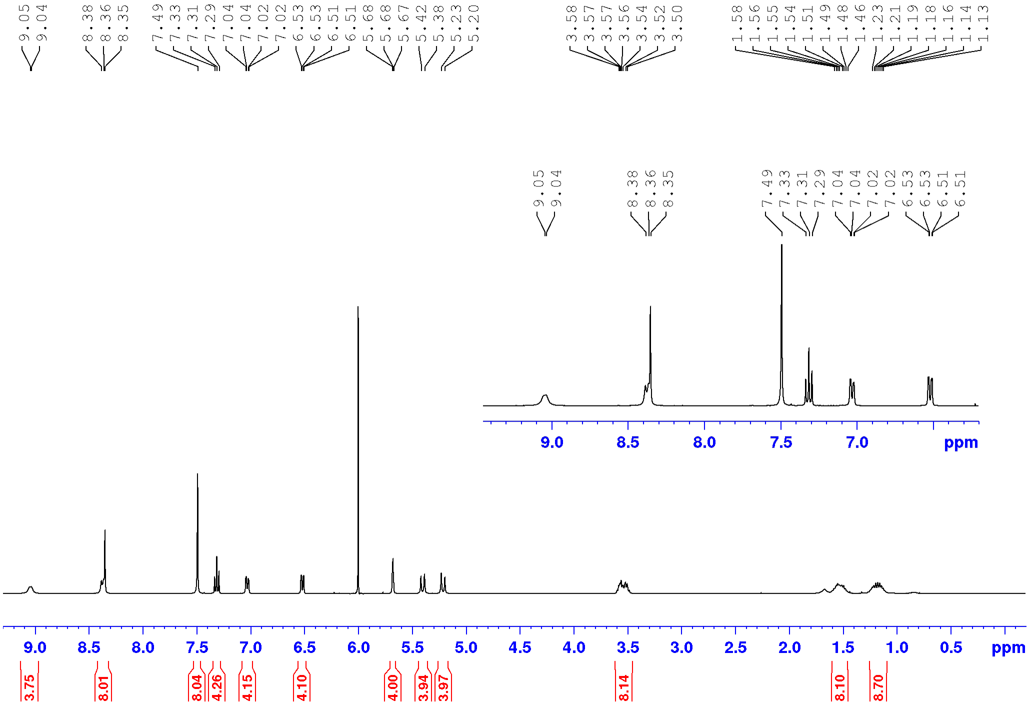

Figure S14. ^{1}H NMR (400 MHz) spectrum of compound **1-*PP*** in TCE-d_2 at 298 K.

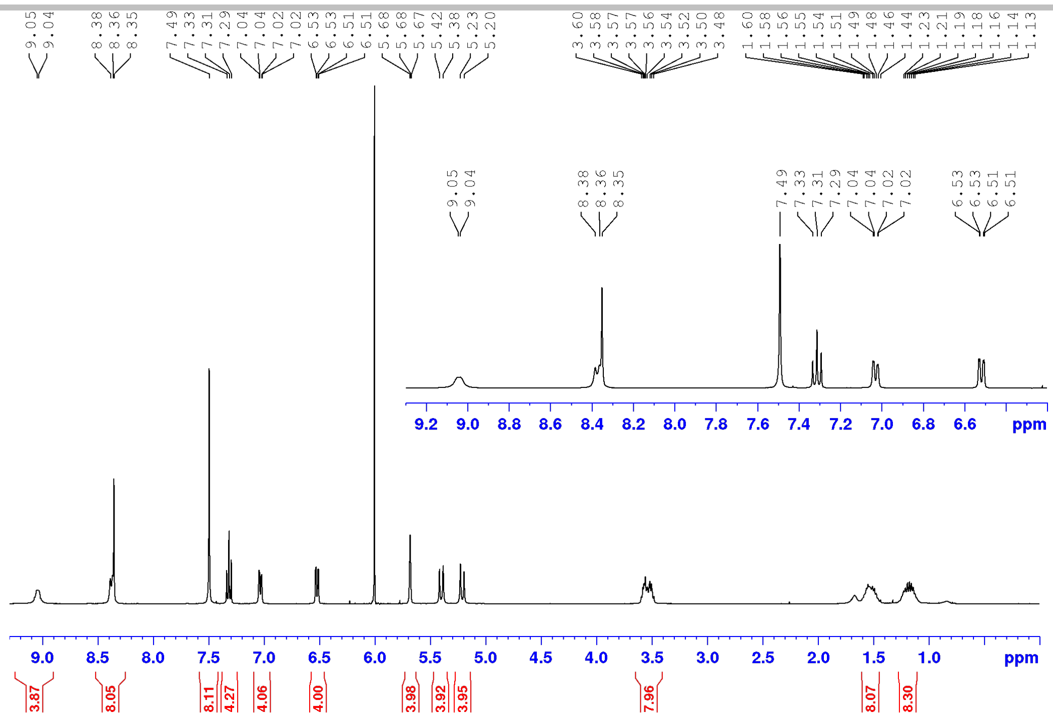

Figure S15. ^{1}H NMR (400 MHz) spectrum of compound **1-*MM*** in TCE-d_2 at 298 K.

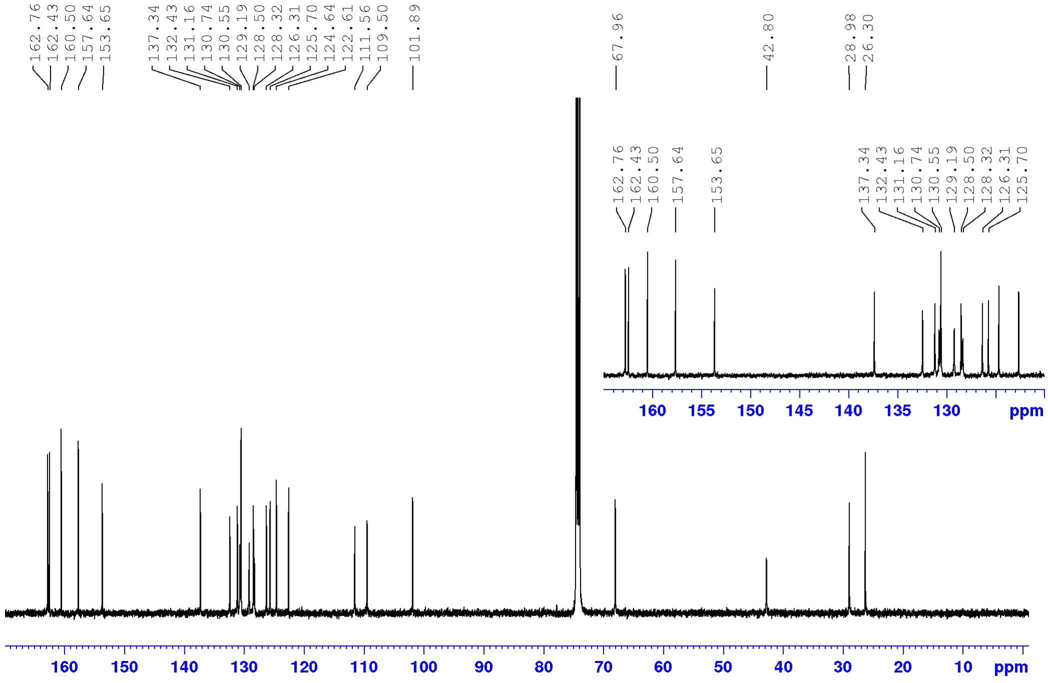

Figure S16. ^{13}C NMR (101 MHz) spectrum of compound **1-*MM*** in TCE-d_2 at 298 K.

Mass Spectra

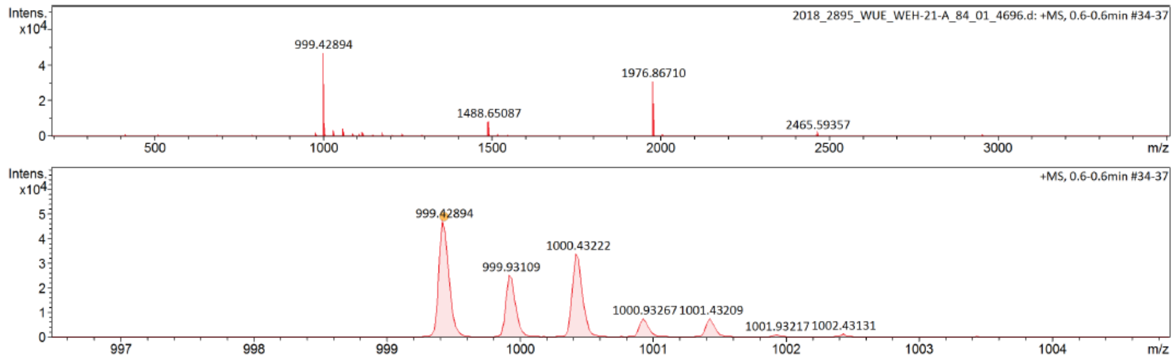

Figure S17. **HRMS** (ESI, positive, acetonitrile/chloroform) of compound **2.**

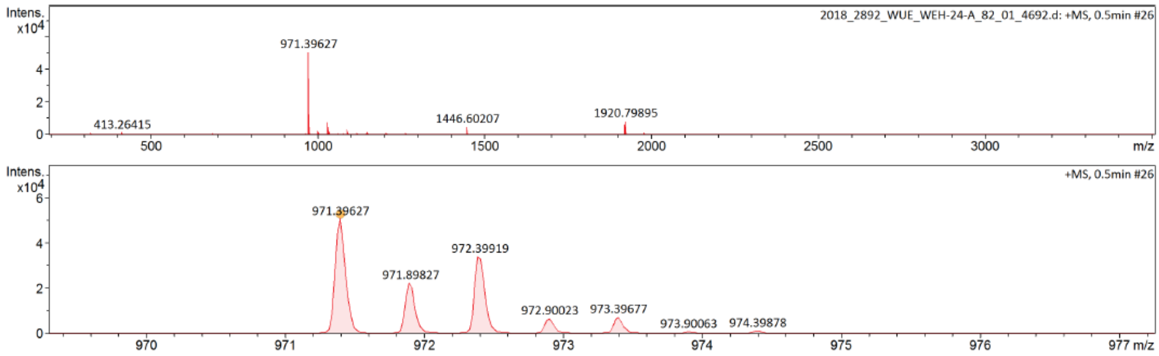

Figure S18. **HRMS** (ESI, positive, acetonitrile/chloroform) of compound **5**.

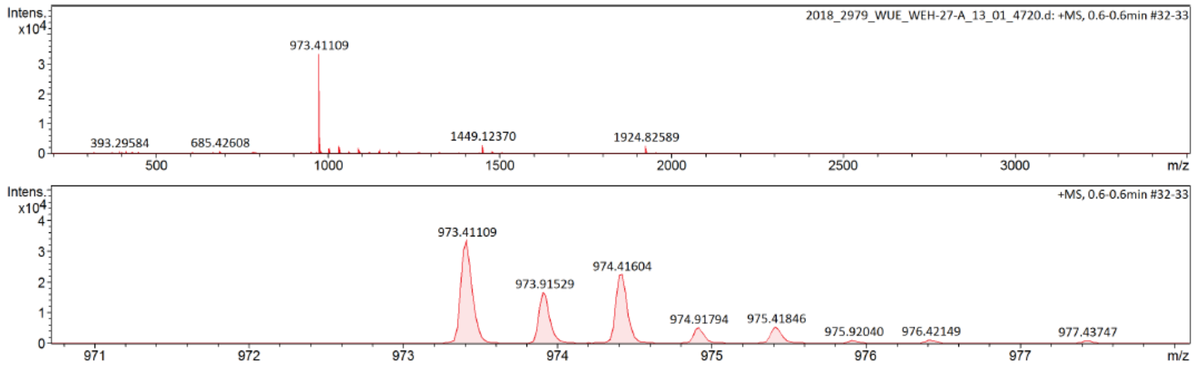

Figure S19. **HRMS** (ESI, positive, acetonitrile/chloroform) of compound **3**.

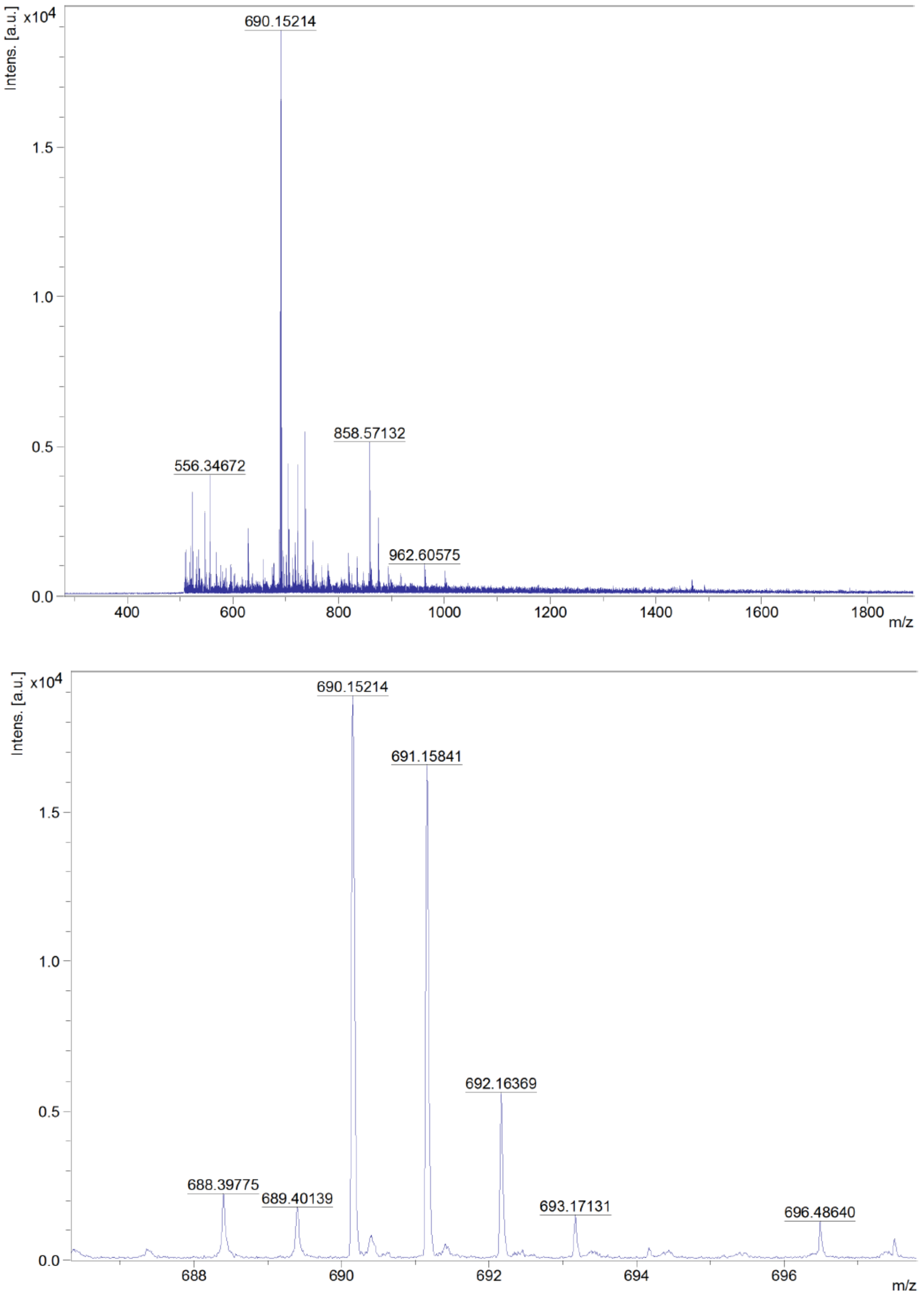

Figure S20. **MALDI-TOF** (positive, DCTB in chloroform) of compound **6**.

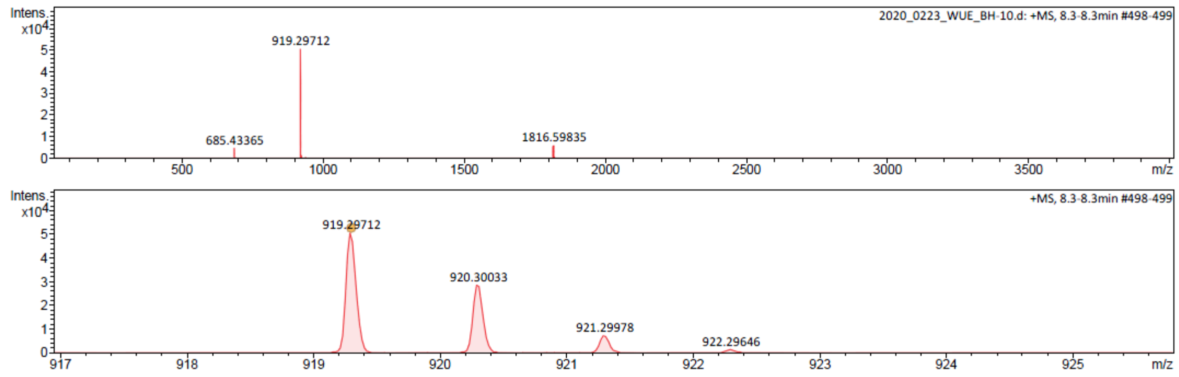

Figure S21. **HRMS** (ESI, positive, acetonitrile/chloroform) of compound **4a** (diastereomeric mixture).

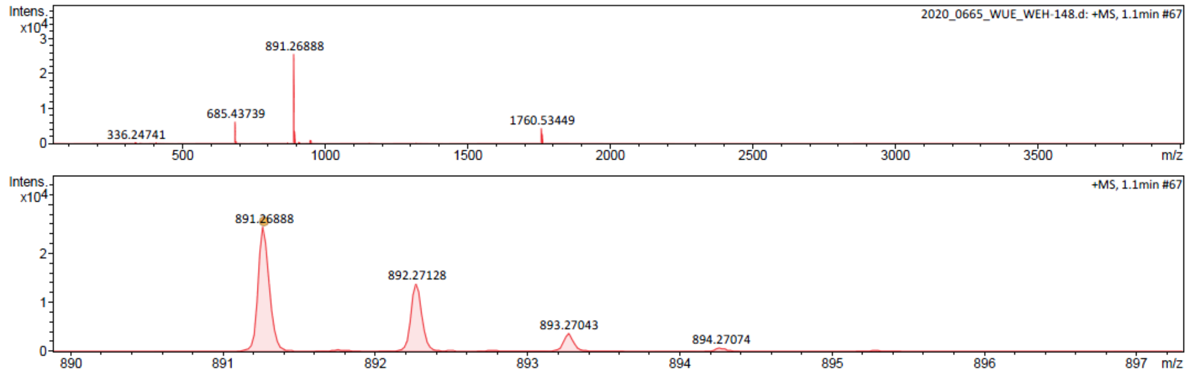

Figure S22. **HRMS** (ESI, positive, acetonitrile/chloroform) of compound **4b**.

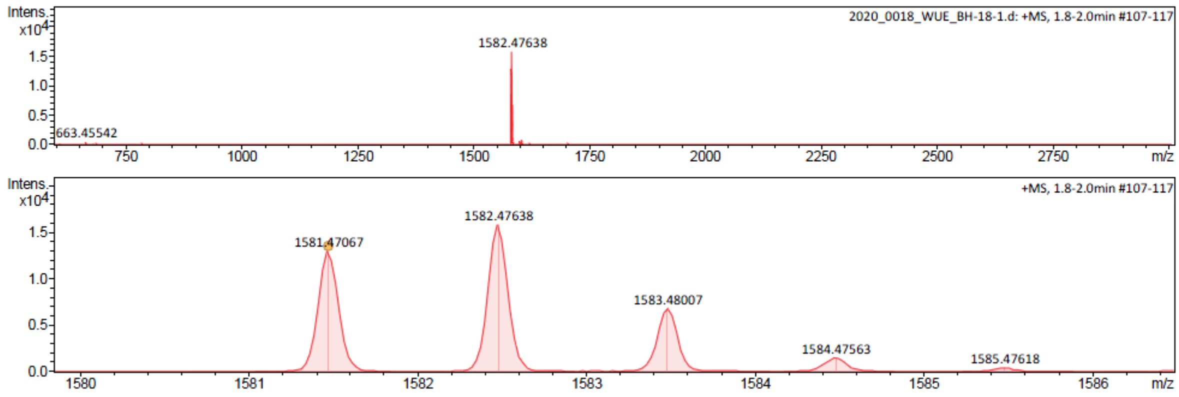

Figure S23. **HRMS** (ESI, positive, acetonitrile/chloroform) of compound **1-*PP*.**

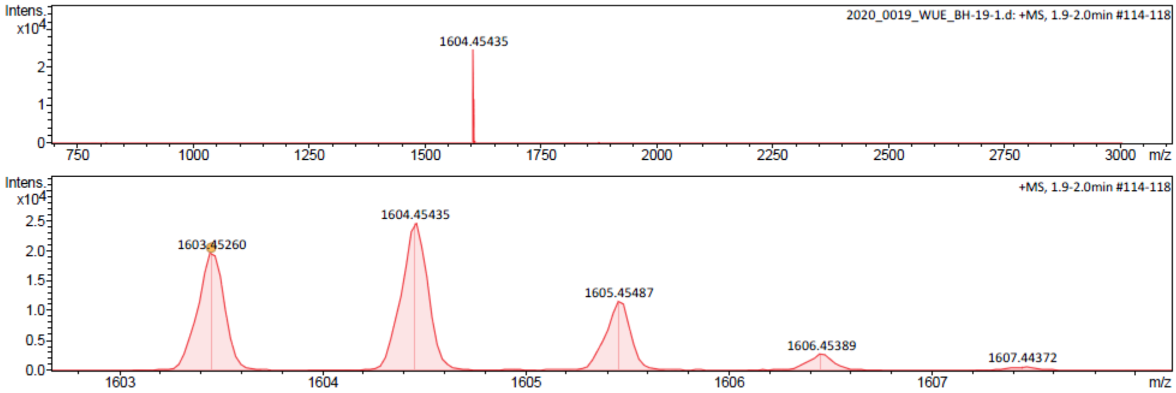

Figure S24. **HRMS** (ESI, positive, acetonitrile/chloroform) of compound **1-*MM***.

Separation of Diastereomers

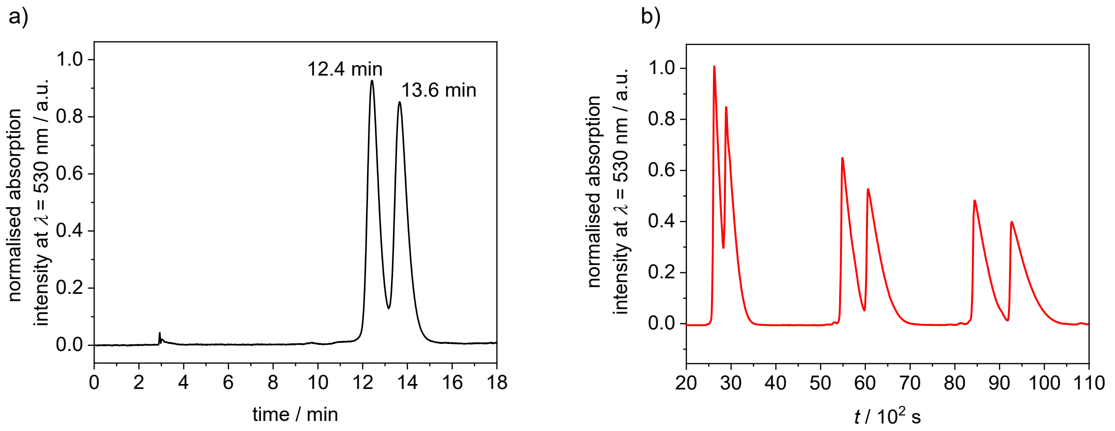

Figure S25. a) Analytical HPLC chromatogram of the separation of **4a-*M*** and **4a-*P*** (flow: 1 mL/min). b) Semipreparative HPLC chromatogram of the separation of **4a-*M*** and **4a-*P*** (flow: 6.5 mL/min). The HPLC was used on recycling mode (three cycles are shown here) with a DCM/*n*-hexane solvent mixture (55:45).

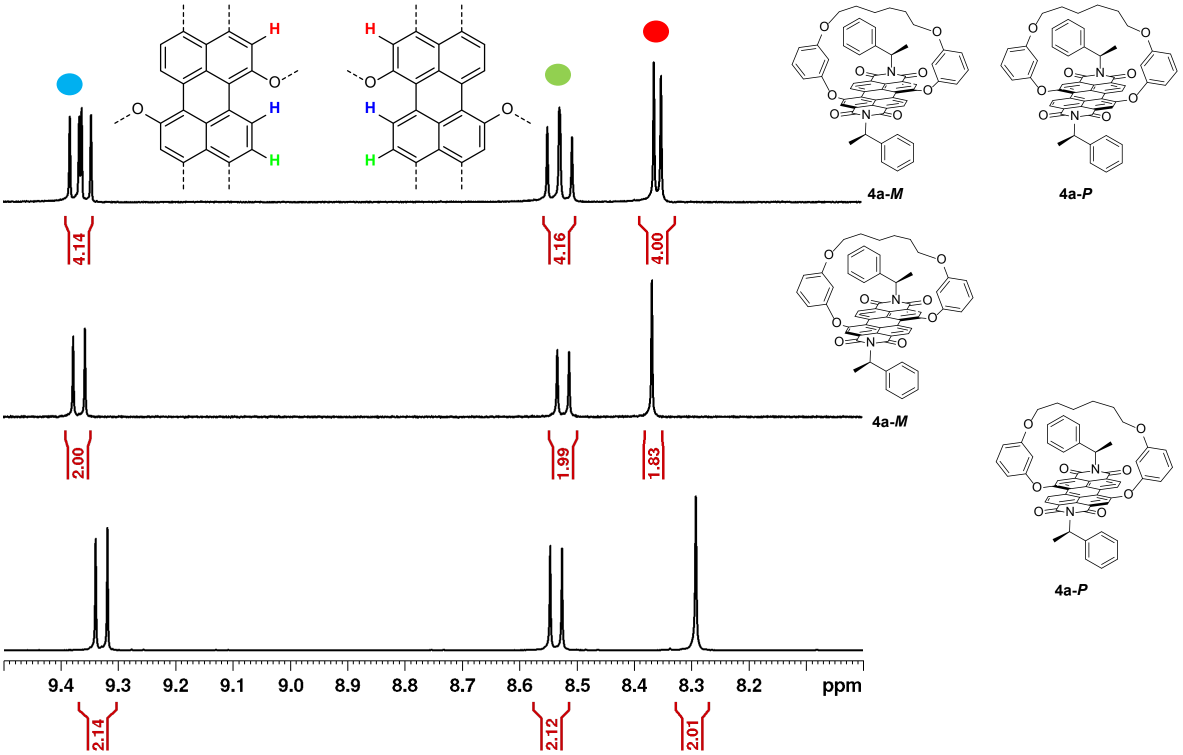

Figure S26. Excerpts of 400 MHz ^{1}H NMR spectrum of the diastereomeric mixture of **4a-*M*** and **4a-*P*** and of the respective isomerically pure compounds after HPLC separation (see Figure S25) at r.t. in $CDCl_3$. Shown are only the perylene protons.

Optical Spectroscopy

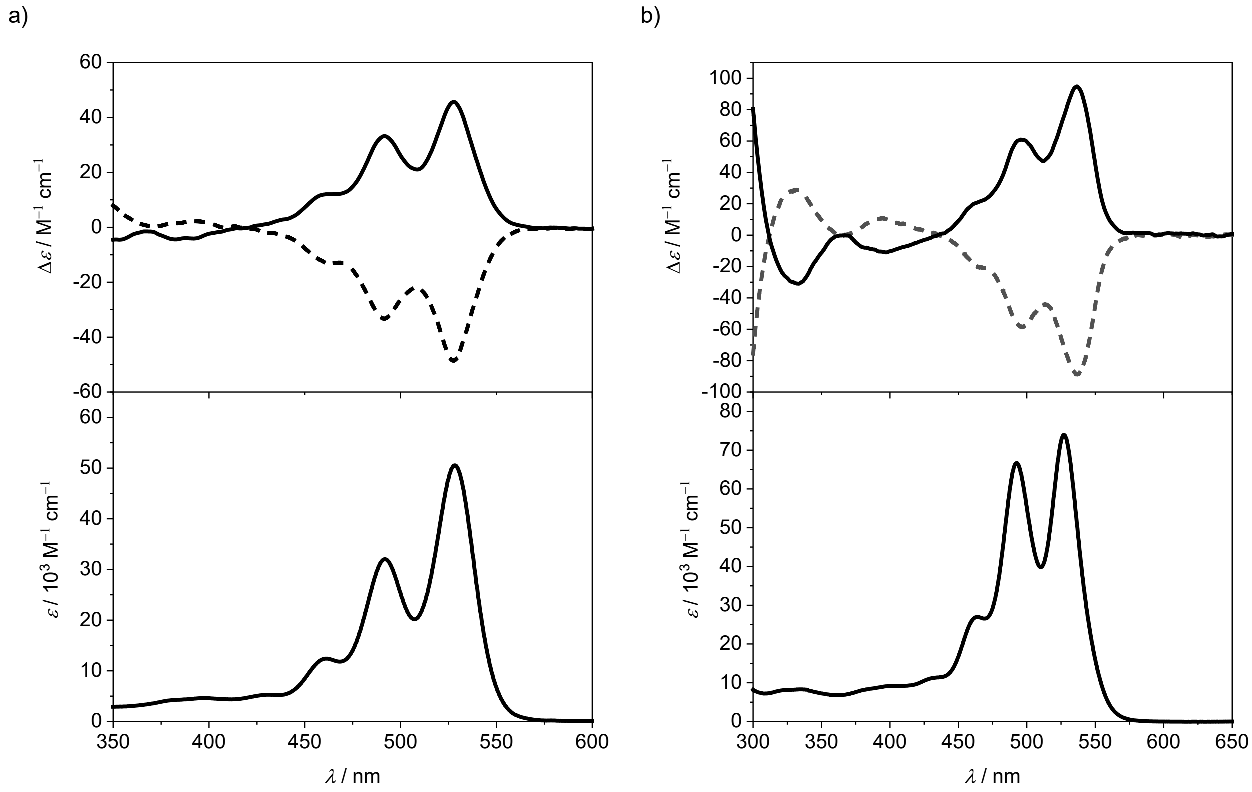

Figure S27. a) CD and UV/vis absorption spectra of **4a-*P*** (solid line) and **4a-*M*** (dashed line) in chloroform at r.t. (c = 30 µM). They behave like pseudo-enantiomers as the imide substituent does not contribute to the optical properties. b) CD and UV/vis absorption spectra of **1-*PP*** (solid lines) and **1-*MM*** (dashed line) in chloroform at r.t. (c = 10 µM).

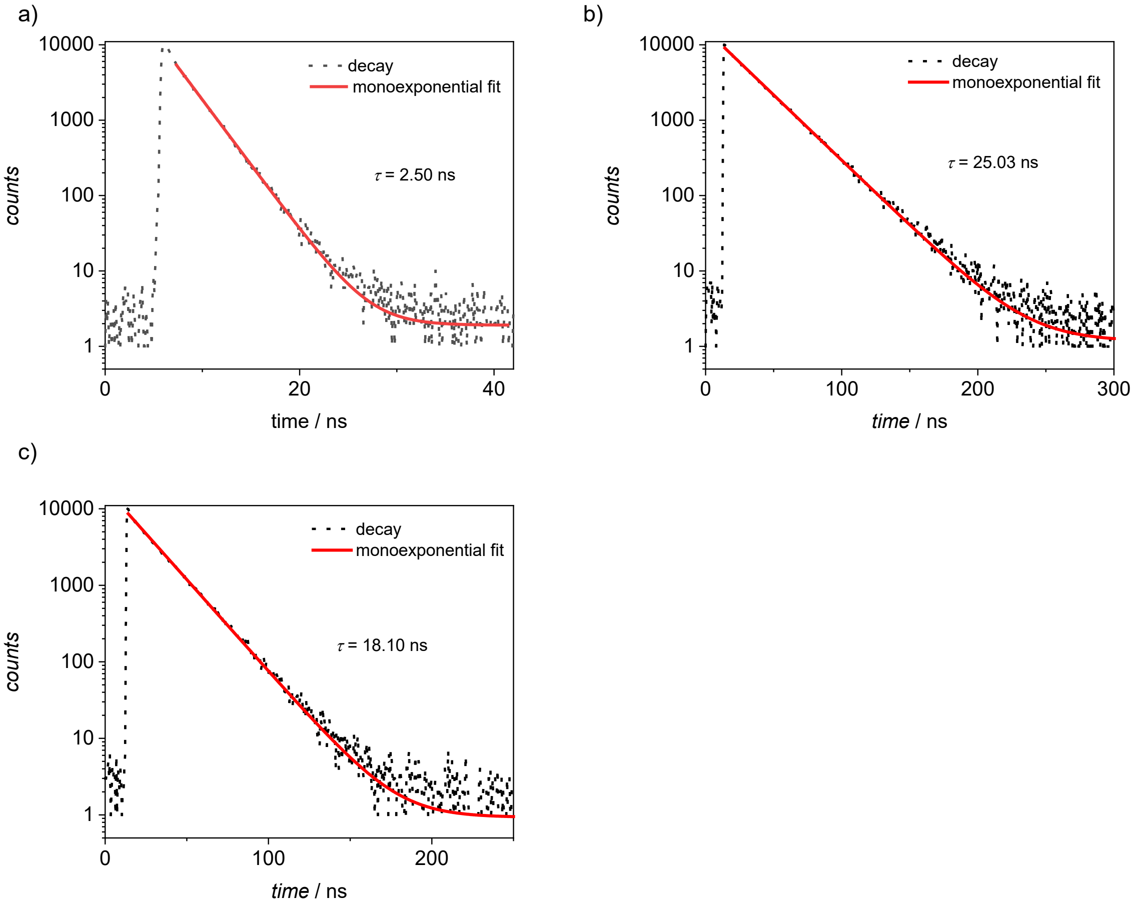

Figure S28. Lifetime measurement (black) of a) free cyclophane **1-*PP*** ($CHCl_3$, 295 K, λ_{ex} = 480 nm, λ_{em} = 545 nm), b) [4]-helicene⊂**1-*PP*** ($CHCl_3$, 295 K, λ_{ex} = 480 nm, λ_{em} = 630 nm), c) [5]-helicene⊂**1-*PP*** ($CHCl_3$, 295 K, λ_{ex} = 480 nm, λ_{em} = 650 nm). The monoexponential fits are shown in red.

Titration Studies

For the titration experiments, a solution of PBI cyclophane **1-*MM*** or **1-*PP*** and the respective guest in excess (see corresponding graphs for exact amount of the individual guest) was titrated to a solution of the pure cyclophane in the same solvent (mixture) of the same concentration keeping the host concentration constant during the experiment. The UV/vis and fluorescence titration data were fitted to a 1:1 binding model.[S8] Note that the conformational lability of [4]-helicene allows a direct titration with the racemic mixture as the corresponding racemization barrier is low enough to allow an instantaneous conformational adaption to the cyclophane receptor while [5]-helicene had to be used enantiomerically pure in order to avoid kinetic effects.

1. Chloroform as solvent

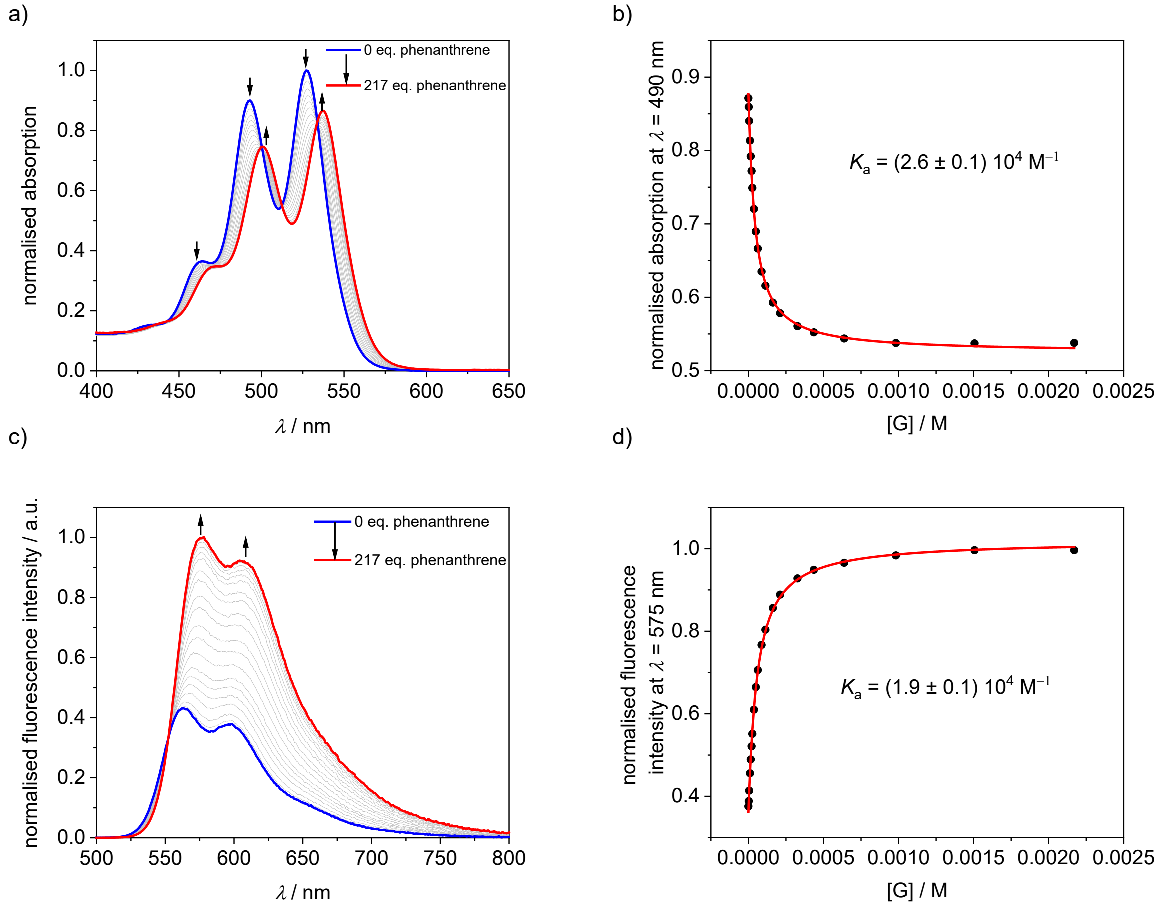

Figure S29. a) UV/vis spectra of cyclophane **1-*PP*** in $CHCl_3$ at 22 °C (c = 10 x 10^{-6} M) upon the addition of phenanthrene as a guest and b) the resulting plot of the absorption at λ = 490 nm with nonlinear curve fit (1:1 binding model, red curve). c) Fluorescence spectra of cyclophane **1-*PP*** in $CHCl_3$ at 22 °C (c = 10 x 10^{-6} M) upon the addition of phenanthrene as a guest and d) the resulting plot of fluorescence intensity at λ = 575 nm with nonlinear curve fit (1:1 binding model, red curve).

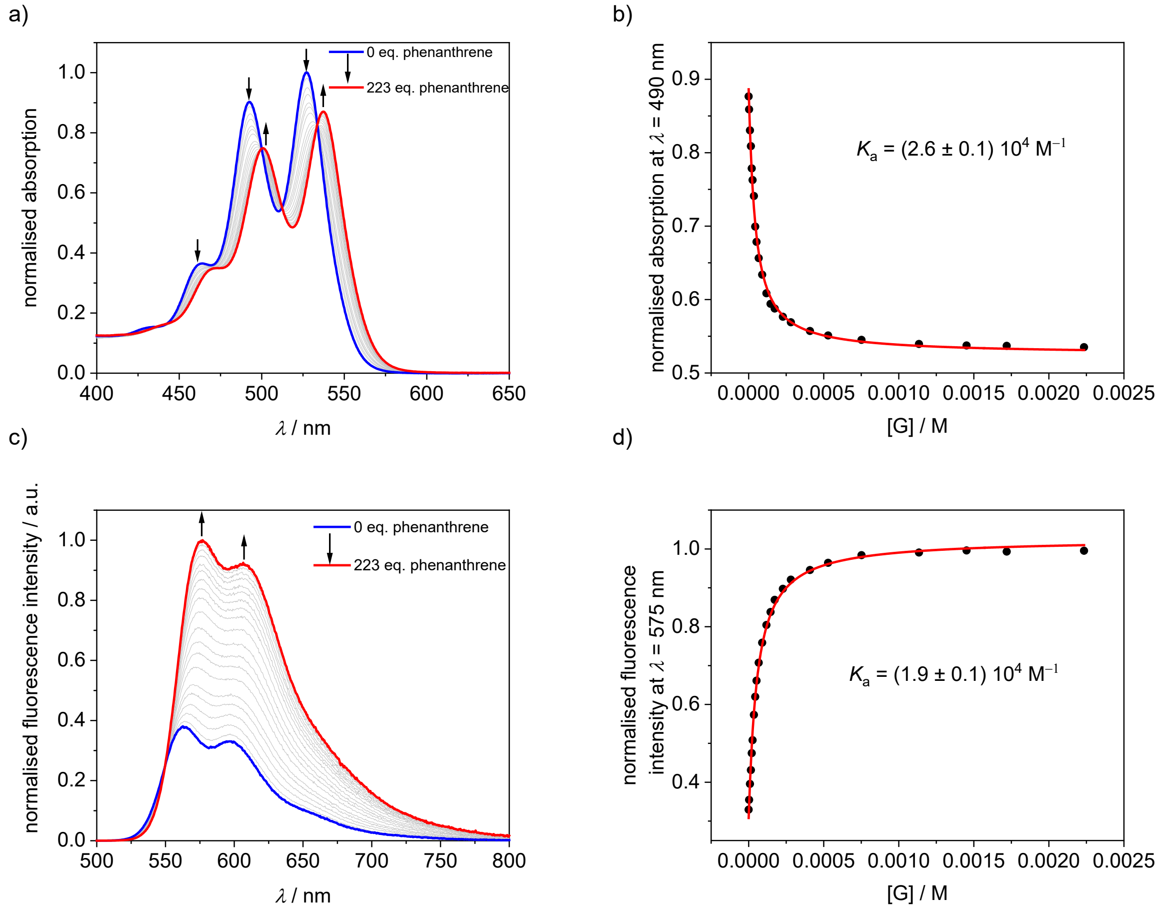

Figure S30. a) UV/vis spectra of cyclophane **1-*MM*** in $CHCl_3$ at 22 °C (c = 10 x 10^{-6} M) upon the addition of phenanthrene as a guest and b) the resulting plot of the absorption at λ = 490 nm with nonlinear curve fit (1:1 binding model, red curve). c) Fluorescence spectra of cyclophane **1-*MM*** in $CHCl_3$ at 22 °C (c = 10 x 10^{-6} M) upon the addition of phenanthrene as a guest and d) the resulting plot of fluorescence intensity at λ = 575 nm with nonlinear curve fit (1:1 binding model, red curve).

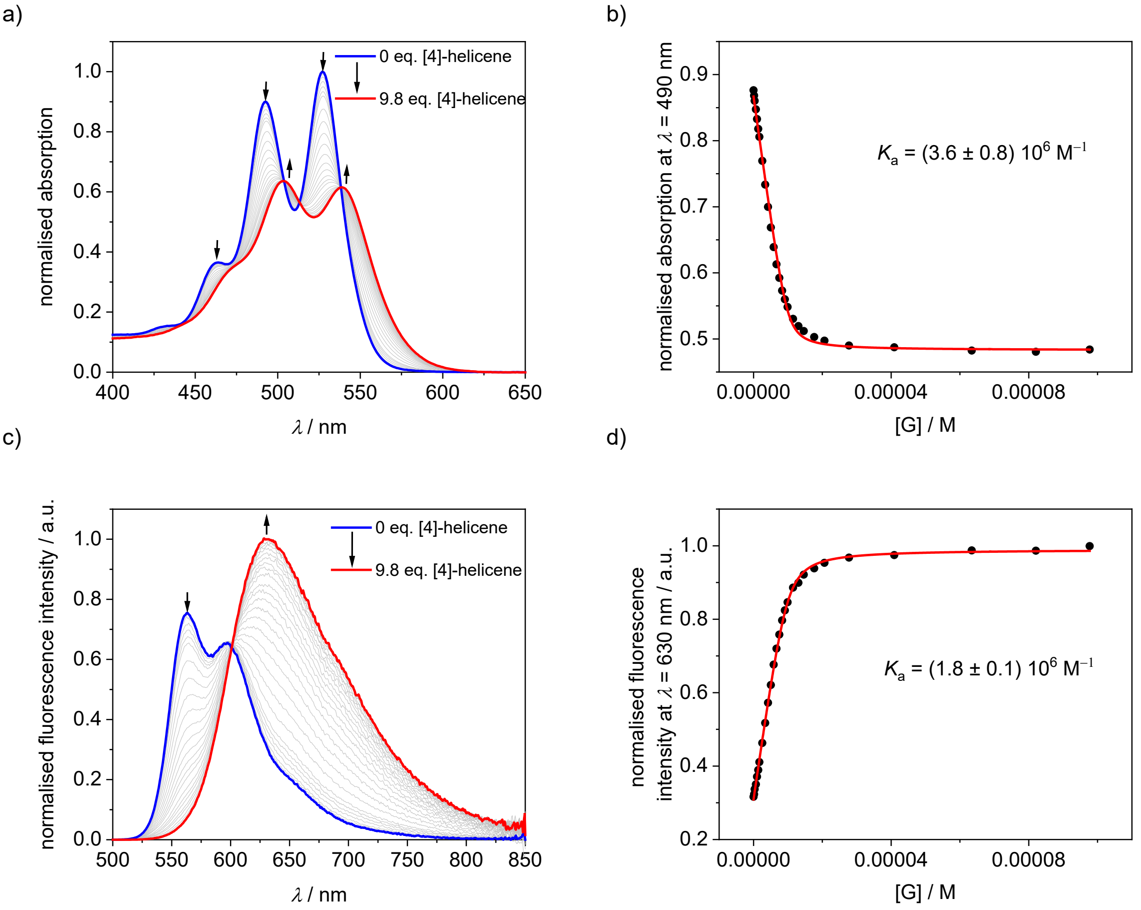

Figure S31. a) UV/vis spectra of cyclophane **1-*PP*** in $CHCl_3$ at 22 °C (*c* = 10 x 10^{-6} M) upon the addition of [4]-helicene as a guest and b) the resulting plot of the absorption at λ = 490 nm with nonlinear curve fit (1:1 binding model, red curve). c) Fluorescence spectra of cyclophane **1-*PP*** in $CHCl_3$ at 22 °C (*c* = 10 x 10^{-6} M) upon the addition of [4]-helicene as a guest and d) the resulting plot of fluorescence intensity at λ = 630 nm with nonlinear curve fit (1:1 binding model, red curve).

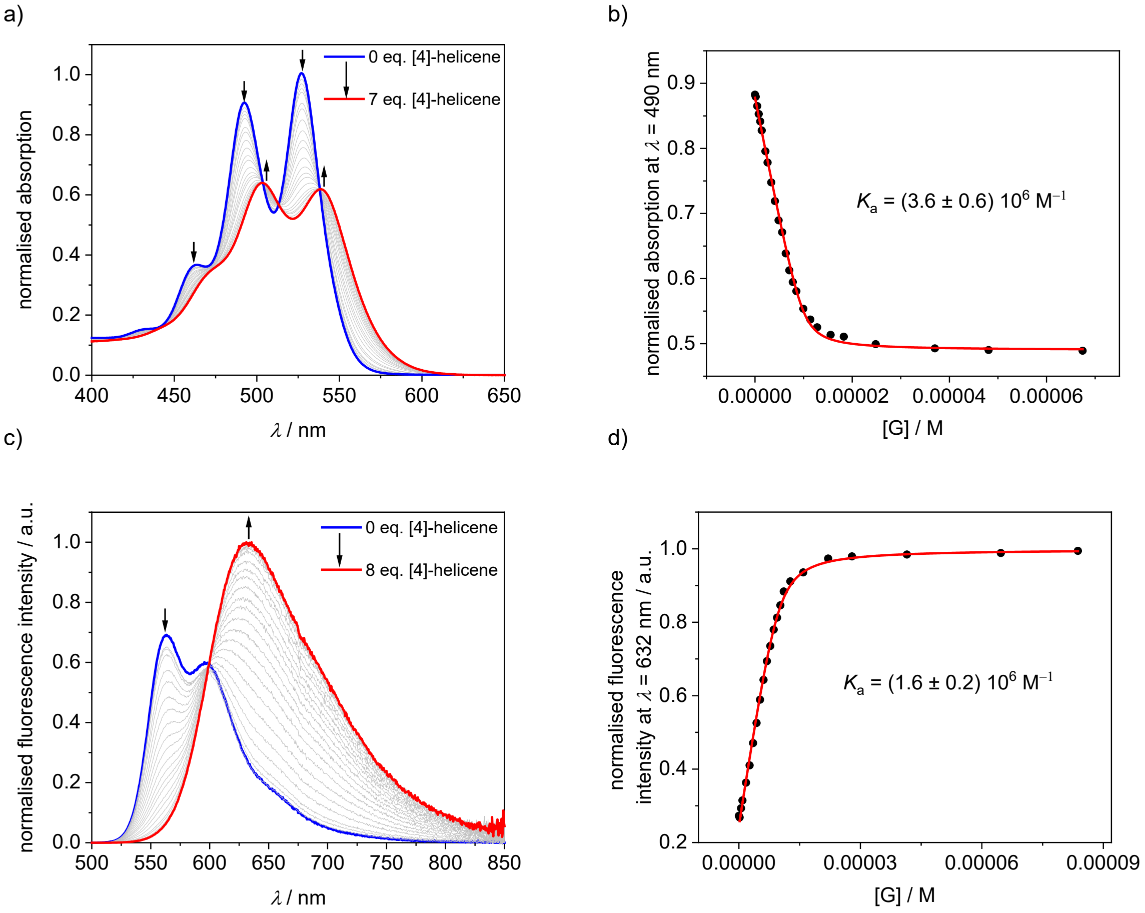

Figure S32. a) UV/vis spectra of cyclophane **1-*MM*** in $CHCl_3$ at 22 °C (c = 10 x 10^{-6} M) upon the addition of [4]-helicene as a guest and b) the resulting plot of the absorption at λ = 490 nm with nonlinear curve fit (1:1 binding model, red curve). c) Fluorescence spectra of cyclophane **1-*MM*** in $CHCl_3$ at 22 °C (c = 10 x 10^{-6} M) upon the addition of [4]-helicene as a guest and d) the resulting plot of fluorescence intensity at λ = 632 nm with nonlinear curve fit (1:1 binding model, red curve).

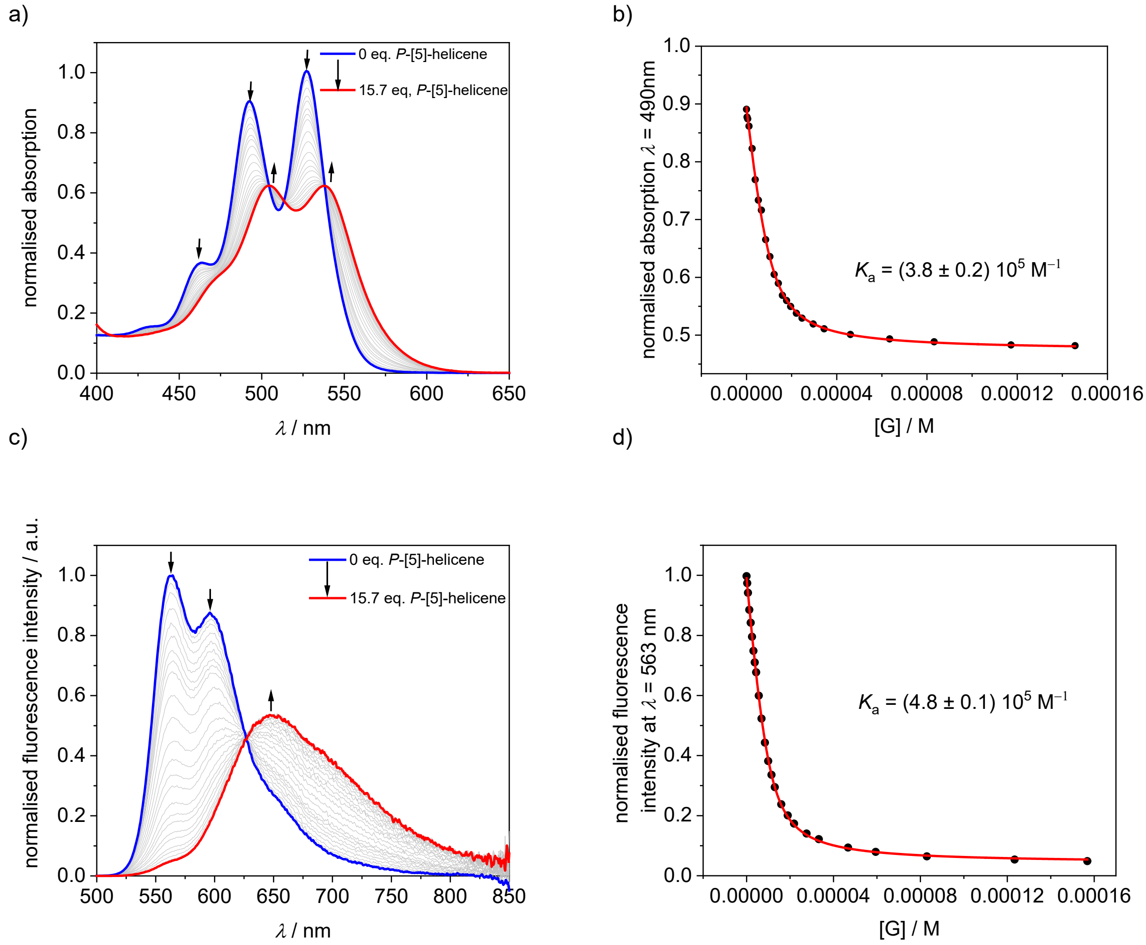

Figure S33. a) UV/vis spectra of cyclophane **1-*PP*** in $CHCl_3$ at 22 °C (c = 10 x 10^{-6} M) upon the addition of *P*-[5]-helicene as a guest and b) the resulting plot of the absorption at λ = 490 nm with nonlinear curve fit (1:1 binding model, red curve). c) Fluorescence spectra of cyclophane **1-*PP*** in $CHCl_3$ at 22 °C (c = 10 x 10^{-6} M) upon the addition of *P*-[5]-helicene as a guest and d) the resulting plot of fluorescence intensity at λ = 563 nm with nonlinear curve fit (1:1 binding model, red curve).

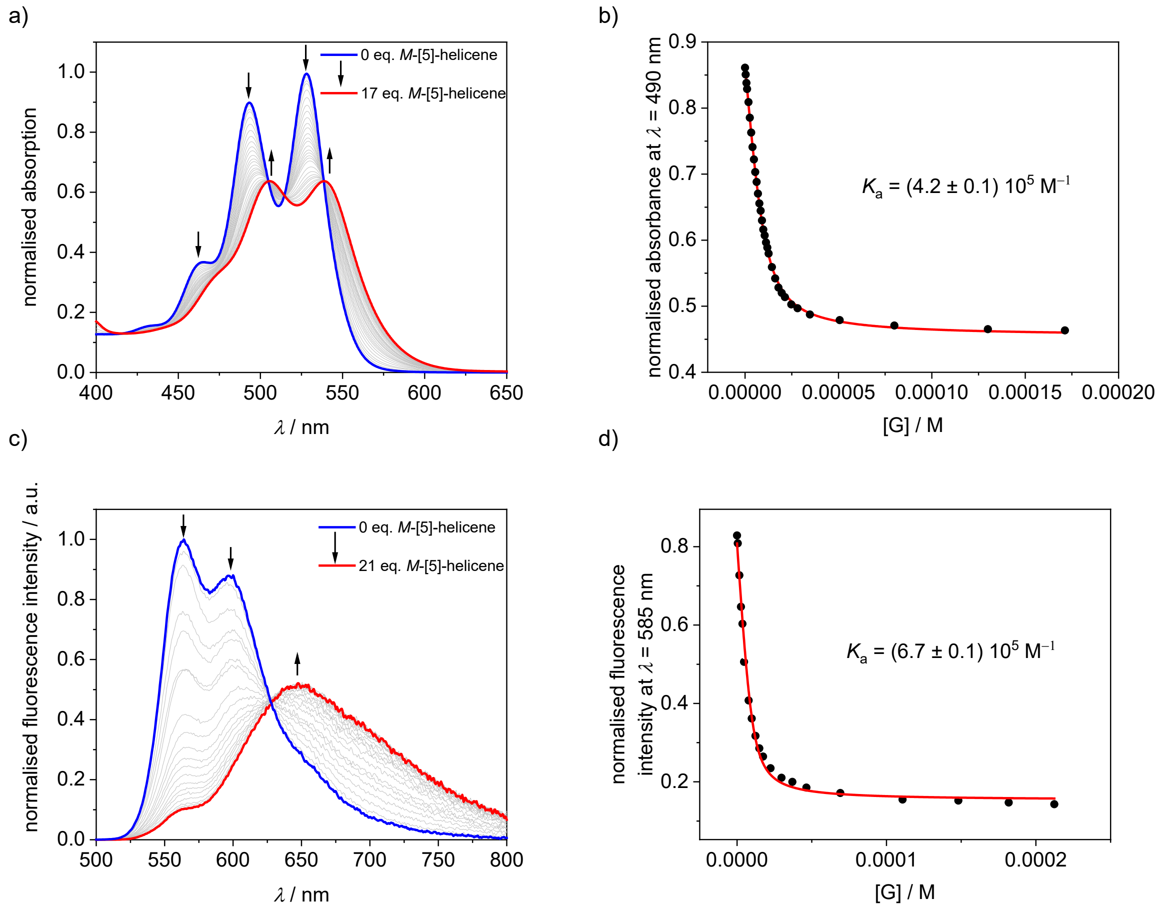

Figure S34. a) UV/vis spectra of cyclophane **1-*MM*** in $CHCl_3$ at 22 °C (c = 10 x 10^{-6} M) upon the addition of *M*-[5]-helicene as a guest and b) the resulting plot of the absorption at λ = 490 nm with nonlinear curve fit (1:1 binding model, red curve). c) Fluorescence spectra of cyclophane **1-*MM*** in $CHCl_3$ at 22 °C (c = 10 x 10^{-6} M) upon the addition of *M*-[5]-helicene as a guest and d) the resulting plot of fluorescence intensity at λ = 585 nm with nonlinear curve fit (1:1 binding model, red curve).

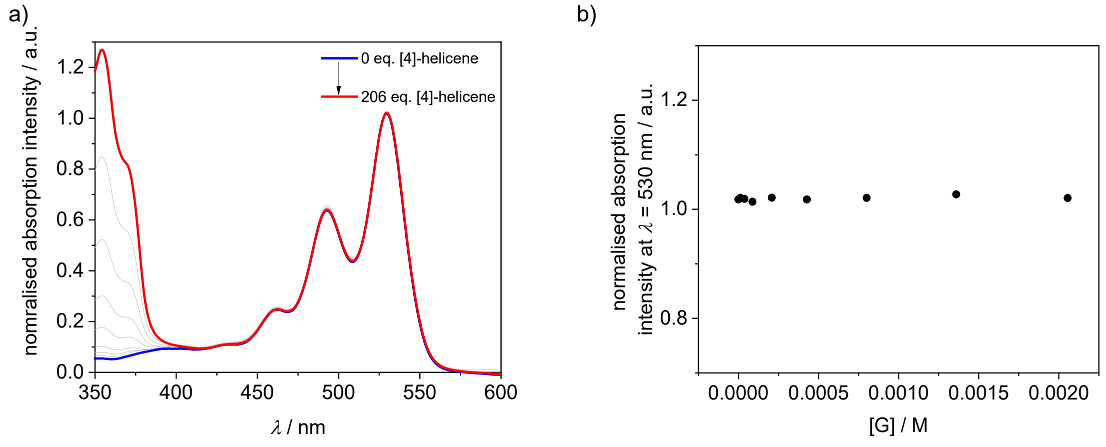

Figure S35. a) UV/vis titration spectra of ***rac*-4b** in $CHCl_3$ at 22 °C (c = 10 x 10^{-6} M) upon the addition of [4]-helicene as a guest and b) the resulting plot of the absorption at λ = 530 nm.

2. Tetrachloromethane as a solvent

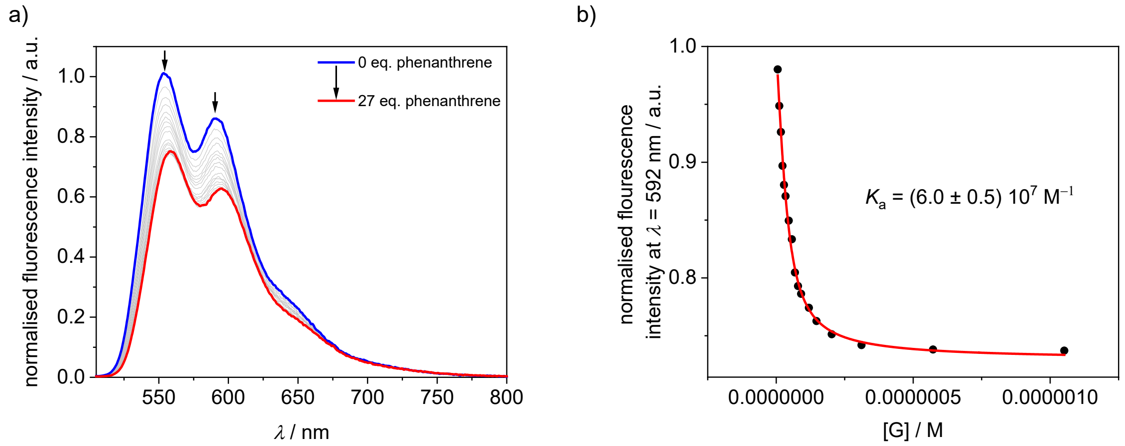

Figure S36. a) Fluorescence spectra of **1-*PP*** in CCl_4 at 22 °C (c = 10 x 10^{-6} M) upon the addition of phenanthrene as a guest and b) the resulting plot of the fluorescence intensity at λ = 592 nm with nonlinear curve fit (1:1 binding model, red curve).

4. Competitive titration studies in tetrachloromethane

For the competitive titration studies a host solution (i.e. **1-*PP***) of a defined concentration was prepared. Phenanthrene, whose binding constant could be determined by direct titration, was added to this solution in excess as a competitive guest. In order to keep the host concentration and the one of phenanthrene constant, the respective guest solution (with the guest of which the binding constant had to be determined) was prepared from the host-phenanthrene-solution. After the stepwise titration, the obtained data were fitted for competitive systems by means of a nonlinear fit.[S9]

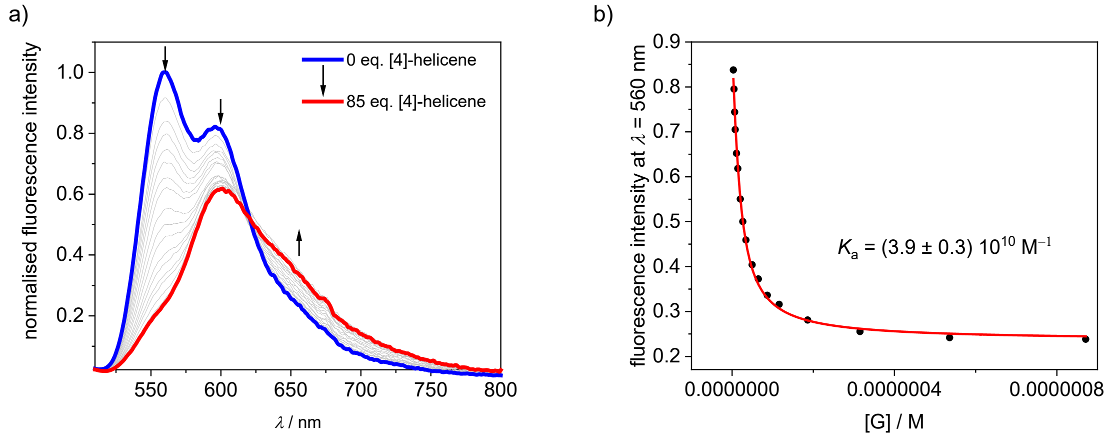

Figure S37. a) Fluorescence spectra of **1-*PP*** (c = 1.3 x 10^{-8} M) in the presence of phenanthrene (c = 8.3 x 10^{-6} M) in CCl_4 at 22 °C upon the addition of [4]-helicene as a guest and b) the resulting plot of the fluorescence intensity at λ = 560 nm with competitive nonlinear curve fit (1:1 binding model, red curve).

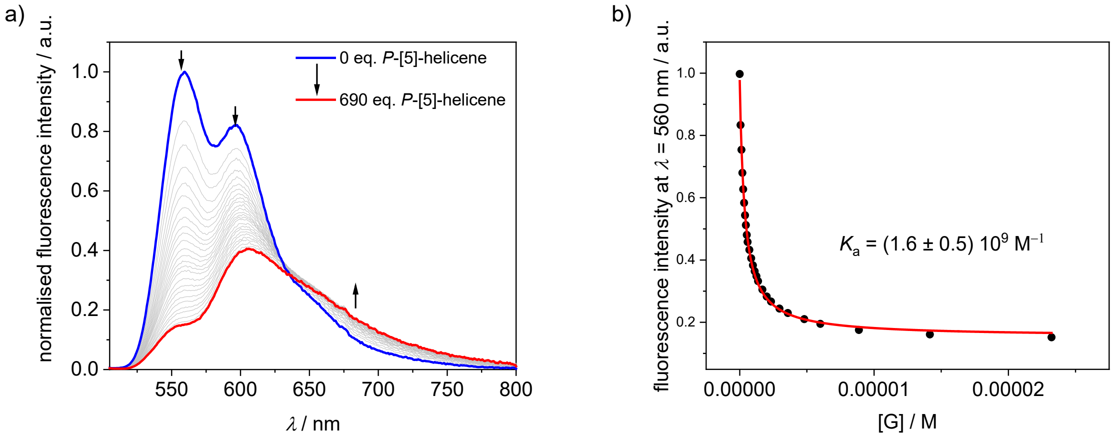

Figure S38. a) Fluorescence spectra of **1-*PP*** (c = 3.4 x 10^{-8} M) in the presence of phenanthrene (c = 9.5 x 10^{-6} M) in CCl_4 at 22°C upon the addition of *P*-[5]-helicene as a guest and b) the resulting plot of the fluorescence intensity at λ = 560 nm with competitive nonlinear curve fit (1:1 binding model, red curve).

DFT Calculations

Energy minimized structures of **4a-*M*** and **4a-*P*** were obtained by DFT calculations (Gaussian 09)[S10] with B3-LYP as functional and 6-31G* as basis set (Figure S39a, b). The optimized structures were used for the calculations of the CD spectra by TD-DFT calculations with CAM-B3-LYP as functional and 6-31G* as basis set (Figure S39c, d). Note that our TD-DFT calculations do not consider vibronic coupling. Therefore, our structures do not reproduce the fine structure present in the experimental spectra.

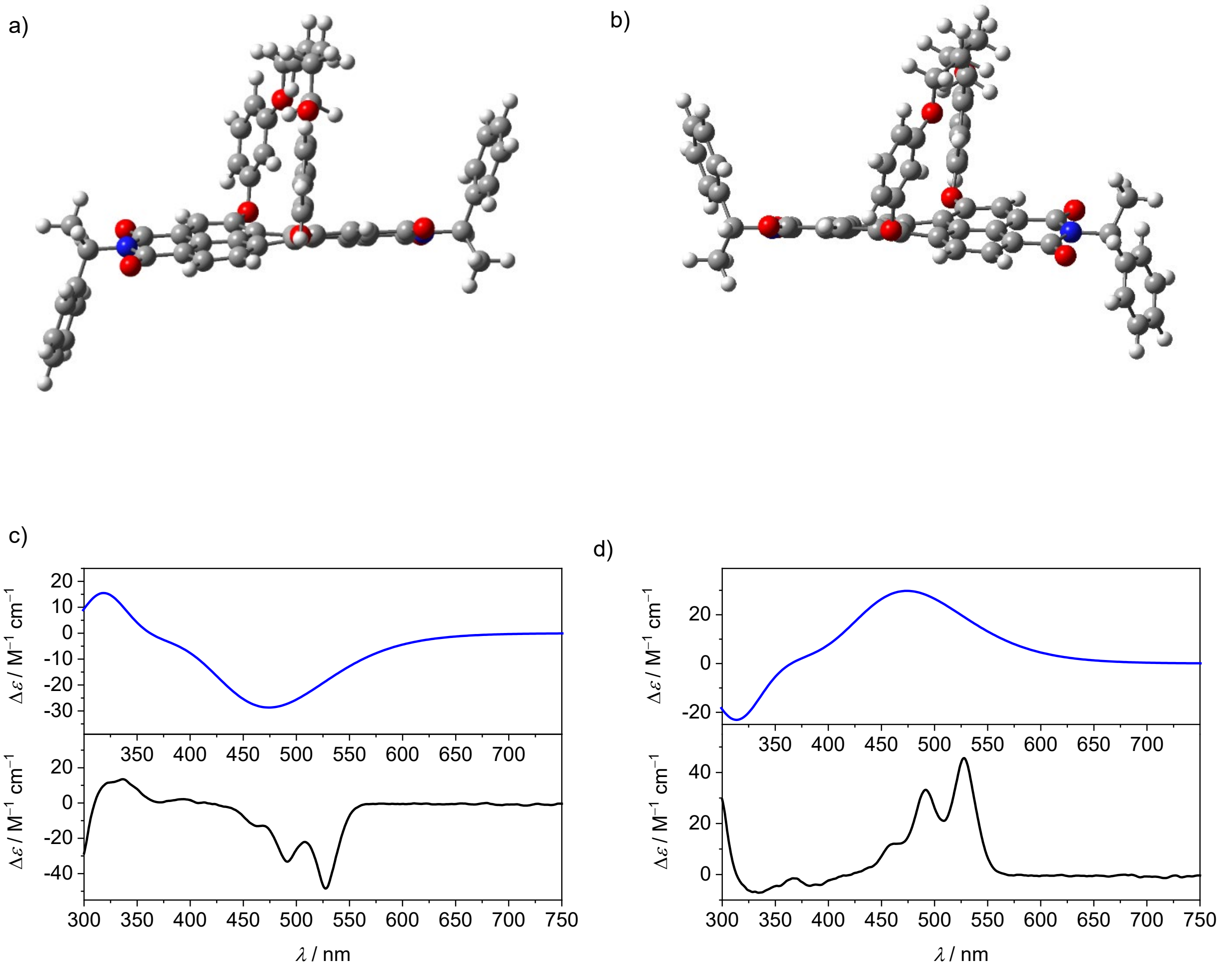

Figure S39. Energy minimized structures of a) **4a-*M*** and b) **4a-*P***. Calculated (blue solid line) and experimental (black solid line) CD spectra of c) **4a-*M*** and d) **4a-*P***.

Energy minimized structures of [4]-helicene⊂**1-*MM*** were obtained by DFT calculations (Gaussian 09)[11] with B3-LYP-D3 as functional and 6-311G* as basis set (Figure S40).

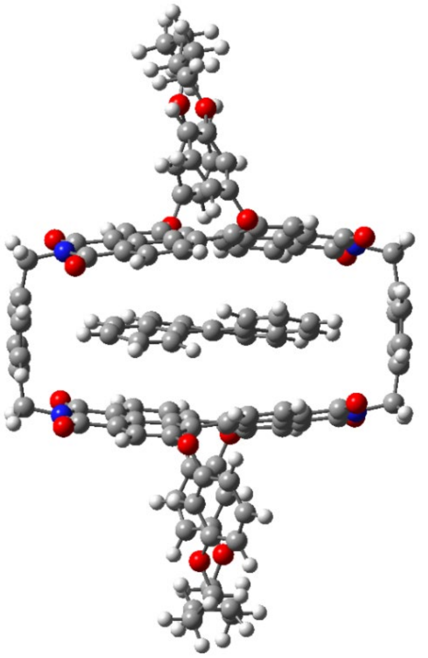

Figure S40. Energy minimized structures of [4]-helicene⊂**1-*MM***.

2D NMR spectra

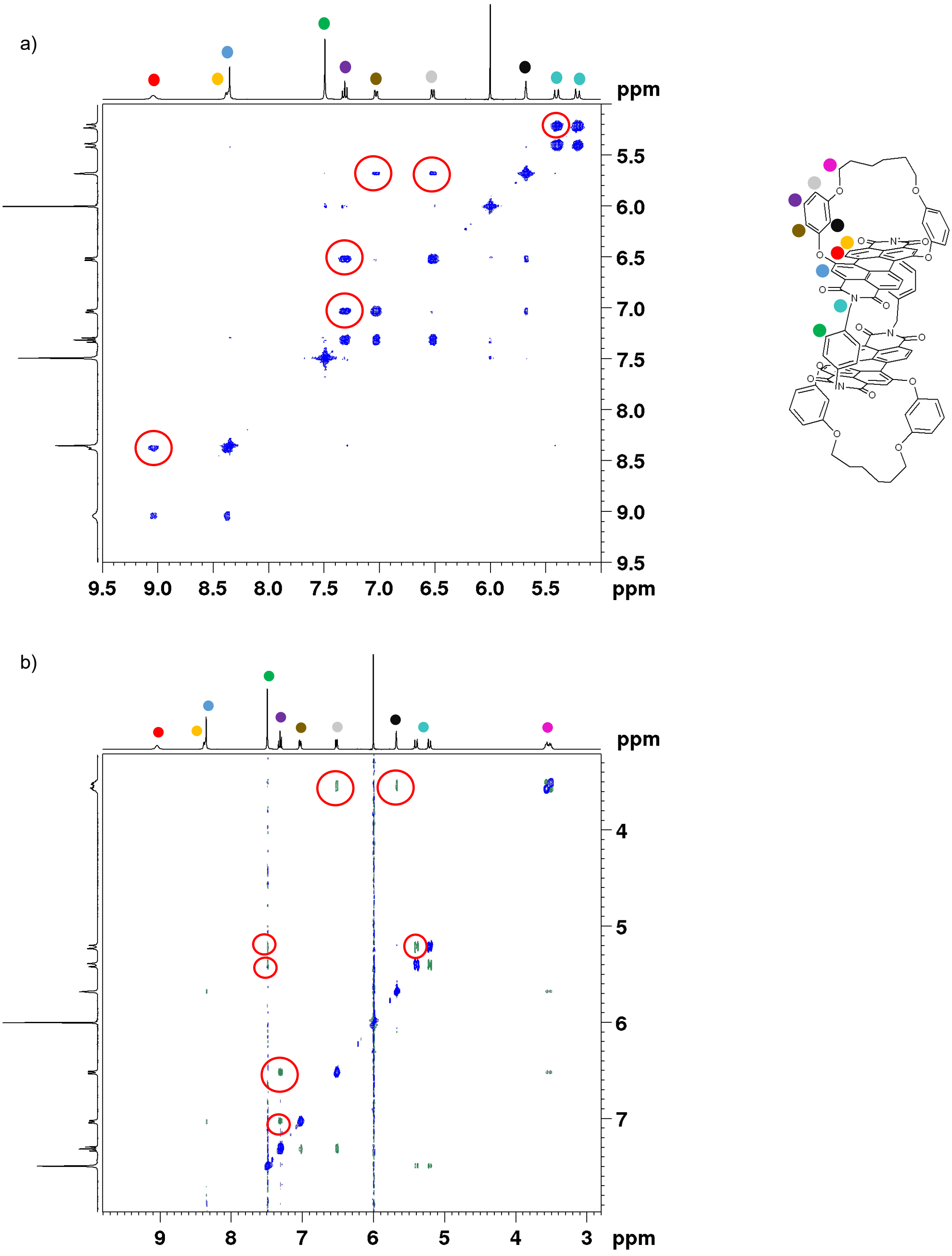

Figure S41. a) ^{1}H-^{1}H COSY NMR spectrum (400 MHz, 298 K) of free **1-*MM*** (*c* = 3.5 mol L^{-1}) in TCE-d_2. The important cross signals are marked in red. b) ^{1}H-^{1}H ROESY NMR spectrum (400 MHz, 298 K) of free **1-*MM*** (*c* = 3.5 mol L^{-1}) in TCE-d_2. The important cross signals are marked in red.

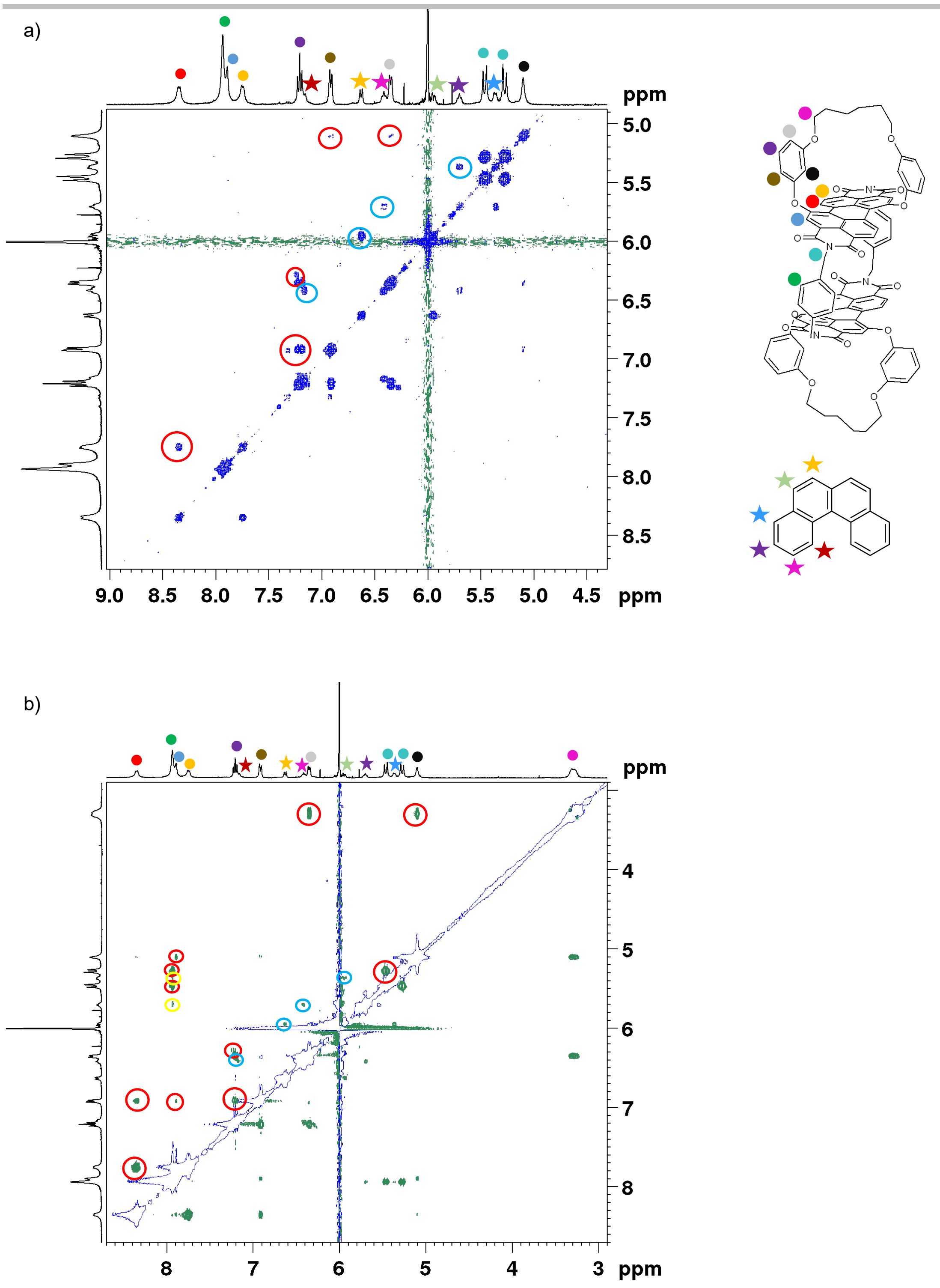

Figure S42. a) ^{1}H-^{1}H COSY NMR spectrum (400 MHz, 298 K) of [4]-helicene⊂**1-*MM*** (c = 3.5 mol L^{-1}) in TCE-d_2. The important cross signals are marked in red (host) and blue (guest). b) ^{1}H-^{1}H ROESY NMR spectrum (400 MHz, 298 K) of [4]-helicene⊂**1-*MM*** (c = 3.5 mol L^{-1}) in TCE-d_2. The important cross signals are marked in red (host), blue (guest) and yellow (host-guest).

Single Crystal X-ray Analysis

The co-crystal of **1-*MM*** and [4]-helicene (~2.5 eq.) was grown in a borosilicate glass tube (10 mm × 75 mm) by slow diffusion of *n*-hexane into chlorobenzene and was obtained as a red, fluorescent block (Figure S43a):

a)

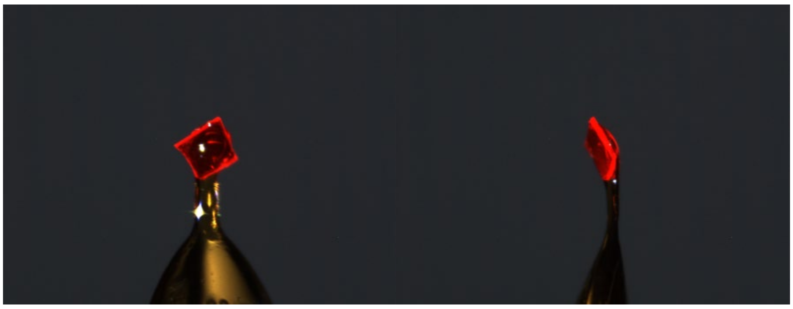

b)

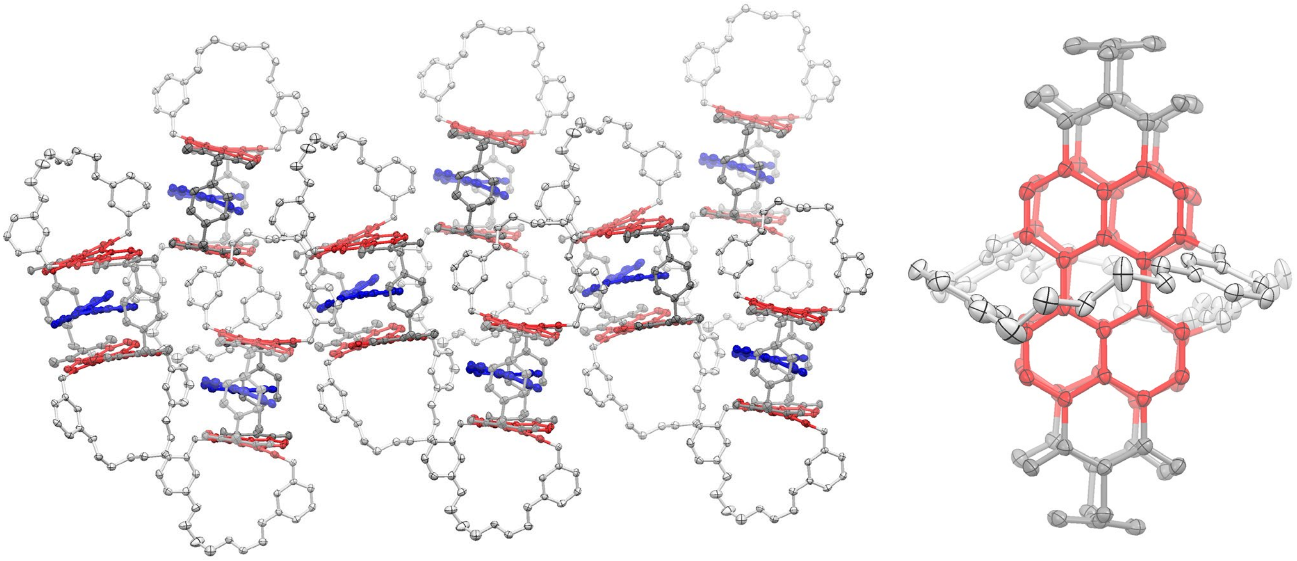

Figure S43. a) Images of the crystals of [4]-helicene⊂**1-*MM***. b) Left: Packing of [4]-helicene⊂**1-*MM*** in the solid state (solvent molecules and hydrogens are omitted for clarity). The perylene unit and the guest are highlighted in red and blue, respectively. Right: Top-view of the cyclophane (guest, solvent molecules and hydrogens are omitted for clarity).

Table S1. Single Crystal X-ray data for [4]-helicene⊂**1**-***MM***.

Compound	[4]-helicene⊂**1**-***MM***
CCDC number	2074804
Sum formula	$C_{154}H_{110}Cl_6N_4O_{16}$
M / g mol^{-1}	2485.16
Crystal size	0.221 x 0.217 x 0.029 mm
Temperature / K	100 (2)
Wavelength / Å	1.54178
Crystal description	Block
Crystal colour	Red
Crystal system	monoclinic
Space group	P 2_1
a / Å	16.9238 (8)
b / Å	18.6773 (9)
c / Å	19.9114 (9)
α / °	90
β / °	108.037 (2)
γ / °	90
V / Å^3	5984.5 (5)
Z	2
ρ_{cal} / g cm^{-3}	1.379
Absorption coefficient / mm^{-1}	1.902
F(000)	2584.0
Measurement range of θ / °	2.334 to 72.489
Limiting indices	–20 <= h <= 20, –23 <= k <= 22, –24 <=l <= 24
Reflections collected / unique	100934 / 23417 [R_{int} = 0.0381]
Completeness / %	100%
Absorption correction	Semi-empirical from equivalents
T_{min}, T_{max}	0.6371, 0.7536
Refinement method	Full-matrix least-squares on F^2
Data / restraints / parameters	23417 / 451 / 1693
Goodness of fit for F^2	1.024
R[I > 2s(I)]	R_1 = 0.0631, wR_2 = 0.1766
R(all data)	R_1 = 0.0668, wR_2 = 0.1811
Largest diff. peak and hole	1.390 and –0.867 e·Å^{-3}

Deracemization Experiments

For a typical deracemization experiments, a solution of **1-*MM*** or **1-*PP*** (63.3 µg, 0.04 µmol) in 4.0 mL chloroform or tetrachloromethane (c = 1.0 x 10^{-5} M) was prepared. Since the solubility in tetrachloromethane is comparatively poor, ultrasonic treatment and heating was used to dissolve the compound. Afterwards, one equivalent of *rac*-[5]-helicene (11.1 µg, 0.04 µmol) was added as a solid to the solution and the time dependent CD measurement was started (Figure S45). When no changes were observed in the CD signal anymore, the formed host-guest complex was separated by GPC (6.5 mL/min, see Figure S44). For the experiments in chloroform, the solution could be directly used for separation by GPC, while in the case of tetrachloromethane the solvent was removed under reduced pressure at r.t.. The residue was then dissolved in chloroform and applied to the GPC separation.
The separated guest was collected and the solvent removed under reduced pressure at r.t.. A CD spectrum of the deracemized guest was measured and a UV/vis absorption spectrum was recorded to determine the concentration from the extinction coefficient, which was determined before from the racemic compound ($\varepsilon_{312\text{ nm}}$ = 31600 M^{-1} cm^{-1}). The enantiomeric excess (*ee*) was calculated by equation S1,[S11] where $\Delta\varepsilon_{312\text{ nm}}$ is the experimental value for $\Delta\varepsilon$ at λ = 312 nm and $\Delta\varepsilon_{312\text{ nm, max}}$ is the value for $\Delta\varepsilon$ at λ = 312 nm for the enantiopure compound.

$$ee = \frac{\Delta\varepsilon_{312\,nm}}{\Delta\varepsilon_{312\,nm,max}} \times 100\% \quad \text{(S1)}$$

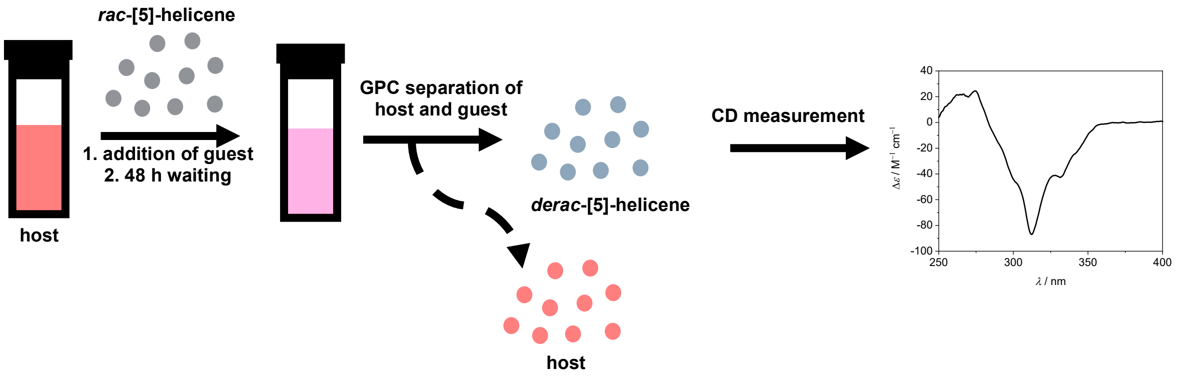

Figure S44. Schematic representation of the experimental protocol for the deracemization experiment of *rac*-[5]-helicene by complexation with **1-*MM*** or **1-*PP***.

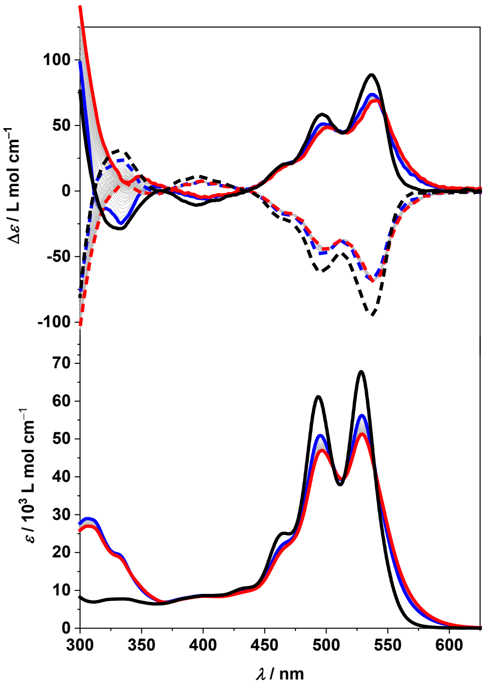

Figure S45. CD (top) and UV/vis (bottom) absorption spectra of **1-*PP*** (solid black line) and **1-*MM*** (dashed black line) and corresponding time dependent spectra after the addition of *rac*-[5]-helicene (blue: first spectrum after the addition, red: after 32 h (no changes after 14 h)) in chloroform at r.t..

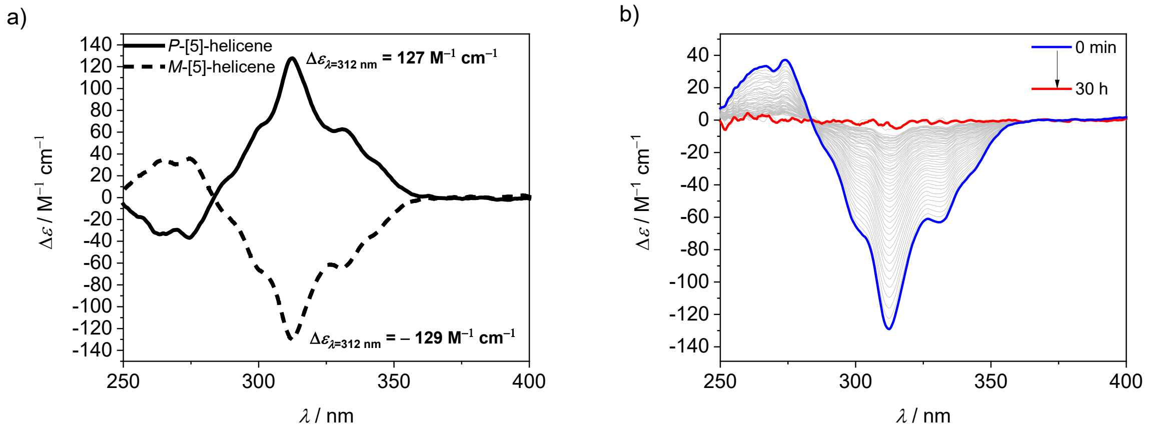

Figure S46. a) CD absorption spectra of *P*-[5]-helicene and *M*-[5]-helicene (*ee* > 98%) in chloroform at 22 °C and b) time dependent CD absorption spectrum of *M*-[5]-helicene in chloroform at 22 °C (right) (*c* = 30 µM).

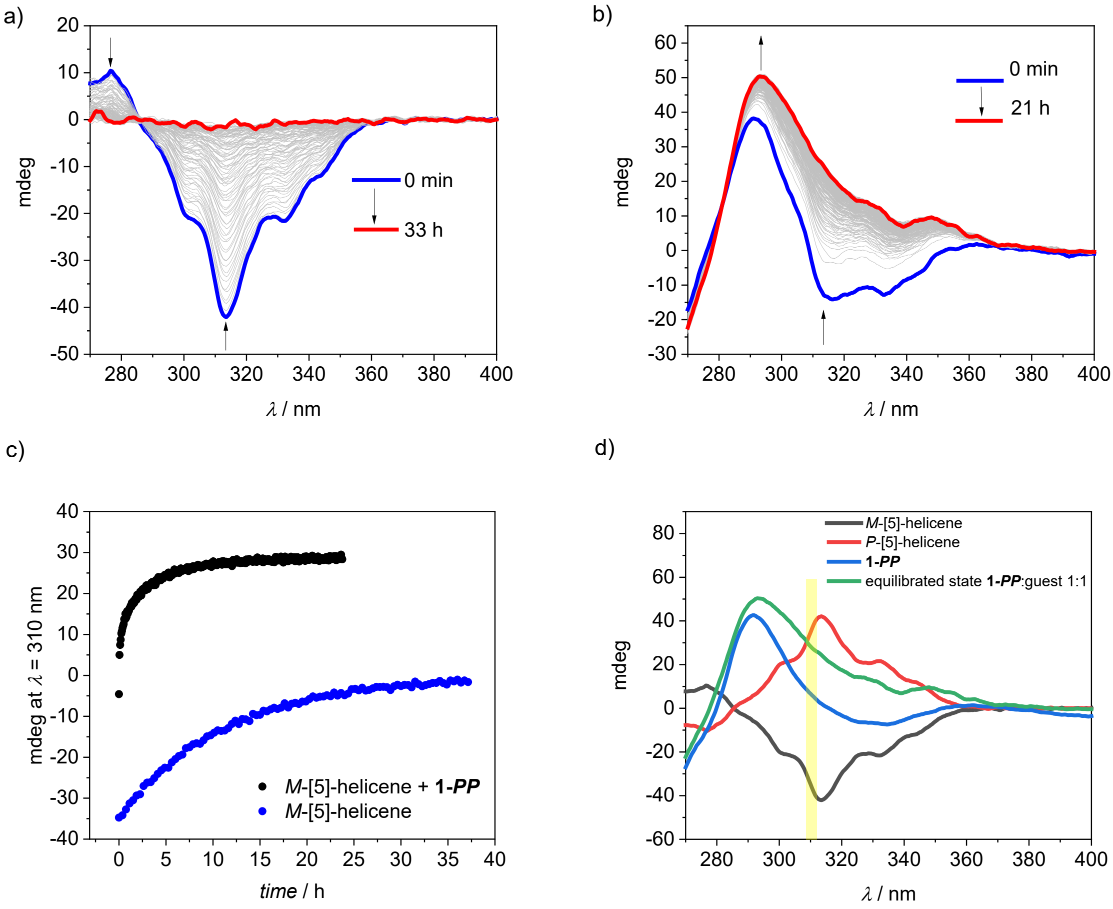

Figure S47. Time dependent CD absorption spectra of a) *M*-[5]-helicene (*ee* > 98%) and b) *M*-[5]-helicene (*ee* > 98%) with one equivalent of **1-*PP*** in tetrachloromethane at 22 °C (c = 10 µM). c) Time course of the CD spectral changes from Figures S46a and S46b at λ = 310 nm. d) CD spectra of the guest, the host and the equilibrated state from figure S 47b in tetrachloromethane at 22 °C (c = 10 µM). At λ = 310 nm (highlighted in yellow) the contribution of **1-*PP*** to the CD spectrum is small (6 mdeg).

References

[S1] S. Sengupta, R.K. Dubey, R. W. M. Hoek, S. P. P. van Eeden, D. D. Gunbaş, F. C. Grozema, E. J. R. Sudhölter, W. F. Jager, *J. Org. Chem.* **2014**, *79*, 6655–6662.
[S2] D. J. Miller, M. Bashir-Uddin Surfraz, M. Akhtar, D. Gani, R. K. Allemann, *Org. Biomol. Chem.* **2004**, *2* , 671–688.
[S3] N. Hoffmann, J.-P Pete, *J. Org. Chem.* **1997**, *62*, 6952–6960.
[S4] A. C. Hernandez-Perez, A. Vlassova, S. K. Collins, *Org. Lett.* **2012,** *14*, 2988–2991.
[S5] A. Brown, C. M. Kemp, S. F. Mason, *J. Chem. Soc. A* **1971**, 751–755.
[S6] G. Seybold, *Dyes Pigm.* **1989**, *11*, 303.
[S7] M. M. Safont-Sempere, P. Osswald, K. Radacki, F. Würthner, *Chem. Eur. J.* **2010,** *16*, 7380–7384.
[S8] M. Sapotta, A. Hofmann, D. Bialas, F. Würthner, *Angew. Chem. Int. Ed.* **2019**, *58*, 3516–3520.
[S9] A. E. Hargrove, Z. Zhong, J. L. Sessler, E. V. Anslyn, *New. J. Chem.* **2010**, *34*, 348–354.
[S10] M. J. Frisch, G. W. Trucks, H. B. Schlegel, G. E. Scuseria, M. A. Robb, J. R. Cheeseman, G. Scalmani, V. Barone, G. A. Petersson, H. Nakatsuji, X. Li, M. Caricato, A. V. Marenich, J. Bloino, B. G. Janesko, R. Gomperts, B. Mennucci, H. P. Hratchian, J. V. Ortiz, A. F. Izmaylov, J. L. Sonnenberg, Williams, F. Ding, F. Lipparini, F. Egidi, J. Goings, B. Peng, A. Petrone, T. Henderson, D. Ranasinghe, V. G. Zakrzewski, J. Gao, N. Rega, G. Zheng, W. Liang, M. Hada, M. Ehara, K. Toyota, R. Fukuda, J. Hasegawa, M. Ishida, T. Nakajima, Y. Honda, O. Kitao, H. Nakai, T. Vreven, K. Throssell, J. A. Montgomery Jr., J. E. Peralta, F. Ogliaro, M. J. Bearpark, J. J. Heyd, E. N. Brothers, K. N. Kudin, V. N. Staroverov, T. A. Keith, R. Kobayashi, J. Normand, K. Raghavachari, A. P. Rendell, J. C. Burant, S. S. Iyengar, J. Tomasi, M. Cossi, J. M. Millam, M. Klene, C. Adamo, R. Cammi, J. W. Ochterski, R. L. Martin, K. Morokuma, O. Farkas, J. B. Foresman, D. J. Fox, Wallingford, CT, **2016**.
[S11] N. Ousaka, S. Yamamoto, H. Iida, T. Iwata, S. Ito, Y. Hijikata, S. Irle, E. Yashima, E., *Nat. Commun.* **2019,** *10*, 1457.
